# A Smartphone App to Improve Medication Adherence in Patients With Type 2 Diabetes in Asia: Feasibility Randomized Controlled Trial

**DOI:** 10.2196/14914

**Published:** 2019-09-12

**Authors:** Zhilian Huang, Eberta Tan, Elaine Lum, Peter Sloot, Bernhard Otto Boehm, Josip Car

**Affiliations:** 1 Centre for Population Health Sciences Lee Kong Chian School of Medicine Nanyang Technological University Singapore Singapore; 2 NTU Institute for Health Technologies Interdisciplinary Graduate School Nanyang Technological University Singapore Singapore; 3 Department of Endocrinology Changi General Hospital Singapore Singapore; 4 Institute of Health and Biomedical Innovation Queensland University of Technology Queensland Australia; 5 School of Clinical Sciences Faculty of Health Queensland University of Technology Queensland Australia; 6 Complexity Institute Nanyang Technological University Singapore Singapore; 7 Institute for Advanced Science University of Amsterdam Amsterdam Netherlands; 8 ITMO University Saint Petersburg Russian Federation; 9 Lee Kong Chian School of Medicine Nanyang Technological University Singapore Singapore; 10 Department of Endocrinology Tan Tock Seng Hospital Singapore Singapore

**Keywords:** smartphone apps, mobile phone apps, medication adherence, type 2 diabetes, feasibility trial, pilot study

## Abstract

**Background:**

The efficacy of smartphone apps for improving medication adherence in type 2 diabetes is not well studied in Asian populations.

**Objective:**

This study aimed to determine the feasibility, acceptability, and clinical outcomes of using a smartphone app to improve medication adherence in a multiethnic Asian population with type 2 diabetes.

**Methods:**

We block randomized 51 nonadherent and digitally literate patients with type 2 diabetes between the ages of 21 and 75 years into two treatment arms (control: usual care; intervention: usual care+Medisafe app) and followed them up for 12 weeks. Recruitment occurred at a public tertiary diabetes specialist outpatient center in Singapore. The intervention group received email reminders to complete online surveys monthly, while the control group only received an email reminder(s) at the end of the study. Barriers to medication adherence and self-appraisal of diabetes were assessed using the Adherence Starts with Knowledge-12 (ASK-12) and Appraisal of Diabetes Scale (ADS) questionnaires at baseline and poststudy in both groups. Perception toward medication adherence and app usage, attitude, and satisfaction were assessed in the intervention group during and after the follow-up period. Sociodemographic data were collected at baseline. Clinical data (ie, hemoglobin A_1c_, body mass index, low-density lipoprotein, high-density lipoprotein, and total cholesterol levels) were extracted from patients’ electronic medical records.

**Results:**

A total of 51 (intervention group: 25 [49%]; control group: 26 [51%]) participants were randomized, of which 41 (intervention group: 22 [88.0%]; control group: 19 [73.1%]) completed the poststudy survey. The baseline-adjusted poststudy ASK-12 score was significantly lower in the intervention group than in the control group (mean difference: 4.7, *P*=.01). No changes were observed in the clinical outcomes. The average 12-week medication adherence rate of participants tracked by the app was between 38.3% and 100% in the intervention group. The majority (>80%) of the participants agreed that the app was easy to use and made them more adherent to their medication.

**Conclusions:**

Our feasibility study showed that among medication-nonadherent patients with type 2 diabetes, a smartphone app intervention was acceptable, improved awareness of medication adherence, and reduced self-reported barriers to medication adherence, but did not improve clinical outcomes in a developed Asian setting.

## Introduction

Medication nonadherence is a complex, costly, and multidimensional problem that involves the patient, his/her health care provider, and the process of taking/using the medication [1]. Patient education, medication management, reminders, and incentives to promote adherence are interventions that have been successful in improving medication adherence worldwide [2]. Despite measures to improve medication adherence, approximately one-third to half of the people with diabetes are still not adherent to their medication [3,4]. People with type 2 diabetes have poorer medication adherence if they do not believe in the safety and efficacy of the medication, which is common in asymptomatic diseases [5]. Poor adherence to diabetes medication results in suboptimal glycemic control [6,7], which increases the risk of diabetes-related complications [8,9], leading to more hospitalization and emergency department visits [10,11].

Smartphone apps are increasingly used as a complementary tool for diabetes self-management (which includes medication management) in recent years. A pooled analysis on the effect of smartphone apps for diabetes self-management found an overall 0.5% reduction in hemoglobin A_1c_ (HbA_1c_) levels [12]. Despite emerging positive evidence on the efficacy of apps in diabetes self-management [13,14], gaps exist in the utility of apps’ features in meeting users’ needs [15-17]. There is a paucity of studies on the efficacy and implementation of smartphone apps in supporting medication taking [12], with only a small number of randomized controlled trials investigating medication adherence in people with high blood pressure [18,19]. Furthermore, diabetes and medication adherence app interventions are not well studied in Asian populations. Asians constitute 60% of people with diabetes globally and are likely to have different cultural beliefs toward disease and medication management [20,21]. This represents missed opportunities to benefit up to 250 million people with diabetes [20]. Given the acceleration of mobile connectivity in the Asia Pacific region in recent years [22], it is important to investigate the receptivity and usage of apps for diabetes medication management in Asian populations with high mobile penetration.

Population-based interventions involving smartphone apps are often complex and multifaceted due to their challenges in controlling the environment [23]. These challenges are amplified when population characteristics are not well understood. In view of the challenges with evaluating complex health interventions, a feasibility and piloting phase to optimize study design and evaluation is warranted [23,24]. We aimed to determine the feasibility, effectiveness, acceptability, and clinical outcomes of using a smartphone app to improve medication adherence in a multiethnic Asian population with type 2 diabetes through a pilot study. Our objectives were to assess the recruitment rate, changes in self-reported barriers to medication adherence, diabetes-related health outcomes, app usage behavior, and satisfaction levels. We referred to the Consolidated Standards of Reporting Trials (CONSORT) eHealth checklist for feasibility trials [25] and the mobile health evidence reporting and assessment (mERA) checklist for mobile health [26] to report the findings of our study.

## Methods

### Study Design

We used a randomized two-arm pre-posttest control group design with a 12-week follow-up period. All participants received usual care, while the intervention group participants additionally downloaded and used the Medisafe app [27] on their personal smartphones during the study.

### Study Setting

Participants were recruited over 10 weeks from September to November 2018 at a tertiary diabetes specialist outpatient center, which is part of a 1000-bed public hospital in (the Eastern region of) Singapore. The center serves subsidized and private patients and nonresidents of Singapore. Patients were self-referred or referred from primary care general practitioners, other departments in the same hospital, or other hospitals. Usual care provided by the center comprises clinic appointments every 3-6 months. At each clinic appointment, patients have their blood pressure and body weight taken, undergo blood tests to monitor their blood glucose and lipid levels, review diabetes management with their endocrinologist, and collect their prescribed medications from the hospital pharmacy. Consultations with the podiatrist, dietitian, or other specialists (ie, ophthalmologist, cardiologist, and renal specialist) were arranged on an ad hoc basis (ie, usually once a year for foot and eye examination). Patients are expected to self-manage their diabetes (following their treatment plan) outside the hospital setting between these scheduled clinic appointments.

Singapore has one of the highest smartphone penetration rates in the world, with 150% mobile subscriptions (one person with two or more mobile subscriptions) and 85% smartphone ownership [28,29].

### Participant Recruitment and Eligibility Criteria

Potential participants were referred by four endocrinologists using a recruitment pamphlet. To be referred by the endocrinologist, participants were at or above the age of 21 years (the legal age for study consent in Singapore), diagnosed with type 2 diabetes according to the American Diabetes Association guidelines, on insulin or oral hypoglycemic agents, and English speakers.

Participants were excluded from the study if they were pregnant, cognitively impaired or diagnosed with psychological issues, prisoners, diagnosed with type 1 diabetes, bed bound and undergoing tube feeding, or prescribed medication for the first time.

Referred patients who consented to participate in the study were asked to complete a baseline questionnaire, which also served as a screening tool to identify eligible patients for randomization. To prevent the “ceiling effect,” participants who were adherent to their medications were screened out of the study. Participants were considered nonadherent to their medication if they answered, “Strongly Agree” or “Agree” to the question, “I forget to take my medicines some of the time” or if they answered “In the last week/month/3 months” to the question, “Have you taken a medicine more or less often than prescribed?”(ask-12-Q8) in the Adherence Starts with Knowledge-12 (ASK-12) questionnaire [30]. To screen participants who were not digitally literate, participants must have responded “Yes” to the question, “Have you used any phone apps in the past 2 weeks?” Lastly, to screen participants who were already using an app to manage their medication, participants must have responded “No” to the question, “Have you used any smartphone app to manage your medications in the past 2 weeks?”

Hence, secondary inclusion criteria for randomization into the study were self-reported medication nonadherence, digital literacy, and nonuse of a medication management app in the past 2 weeks.

### Study Procedures

Patients with type 2 diabetes attending their scheduled clinic appointments, who met the referral inclusion criteria, were referred to the researchers by their endocrinologist. Interested patients proceeded to provide informed consent. The patient was termed a research participant once the informed consent document was signed. At the point of consent seeking, researchers explained to potential participants that they may or may not be selected for the study, depending on their eligibility, which can only be determined after they respond to the baseline questionnaire. Informed consent was collected with printed hardcopy forms.

Study data were collected and managed using Research Electronic Data Capture (REDCap) by Nanyang Technological University [31,32]. REDCap is a secure, Web-based software platform designed to support data capture for research studies, providing an intuitive interface for validated data capture; audit trails for tracking data manipulation and export procedures; automated export procedures for seamless data downloads to common statistical packages; and procedures for data integration and interoperability with external sources. Upon receiving informed consent, REDCap generates a unique survey code linked to each participant’s predefined email for the baseline and subsequent follow-up surveys. All data in REDCap are deidentified apart from participants’ email address. Baseline data were collected using an iPad on the day of recruitment prior to randomization.

### Intervention

The intervention group participants were asked to download and use the Medisafe app to help them manage their medications for 12 weeks. Participants were assisted by the researchers to download the Medisafe app on their personal smartphone, to set their medication schedule and reminder on the app, and to use the app. Participants were asked to use the app freely outside the health care setting and add the research group as a “Medfriend” for their medication-taking patterns to be observed.

Medisafe is a commercial, free medication management app available on both Android and iOS platforms. Its features include medication scheduling, reminder, tracking, data sharing, and medication adherence assessments. We selected a commercial app with evidence supporting its effectiveness [13,33] to assess the feasibility of a smartphone app in promoting medication adherence in patients with type 2 diabetes.

The intervention group participants were reminded via email to complete two intermediate and one final online survey at 4-week intervals during the 12-week follow-up period. Control group participants were instructed to complete only one online survey at the end of the 12-week follow-up period. All follow-up surveys were conducted online via a unique link sent to the participants’ email address. Each unique survey link was accessible for a maximum of 14 days or until the participant completed the survey. Participants in both groups were reminded by calling them on their mobile phone to complete the final survey if no response was received a week after the survey was sent out. Participants were given supermarket vouchers on completion of each online survey. Voucher rewards were consolidated and collected from the diabetes center by participants at the end of the study. We collected some participant feedback with the online satisfaction survey and while handing out vouchers to participants who completed the online survey(s).

### Outcomes

**Figure 1:**
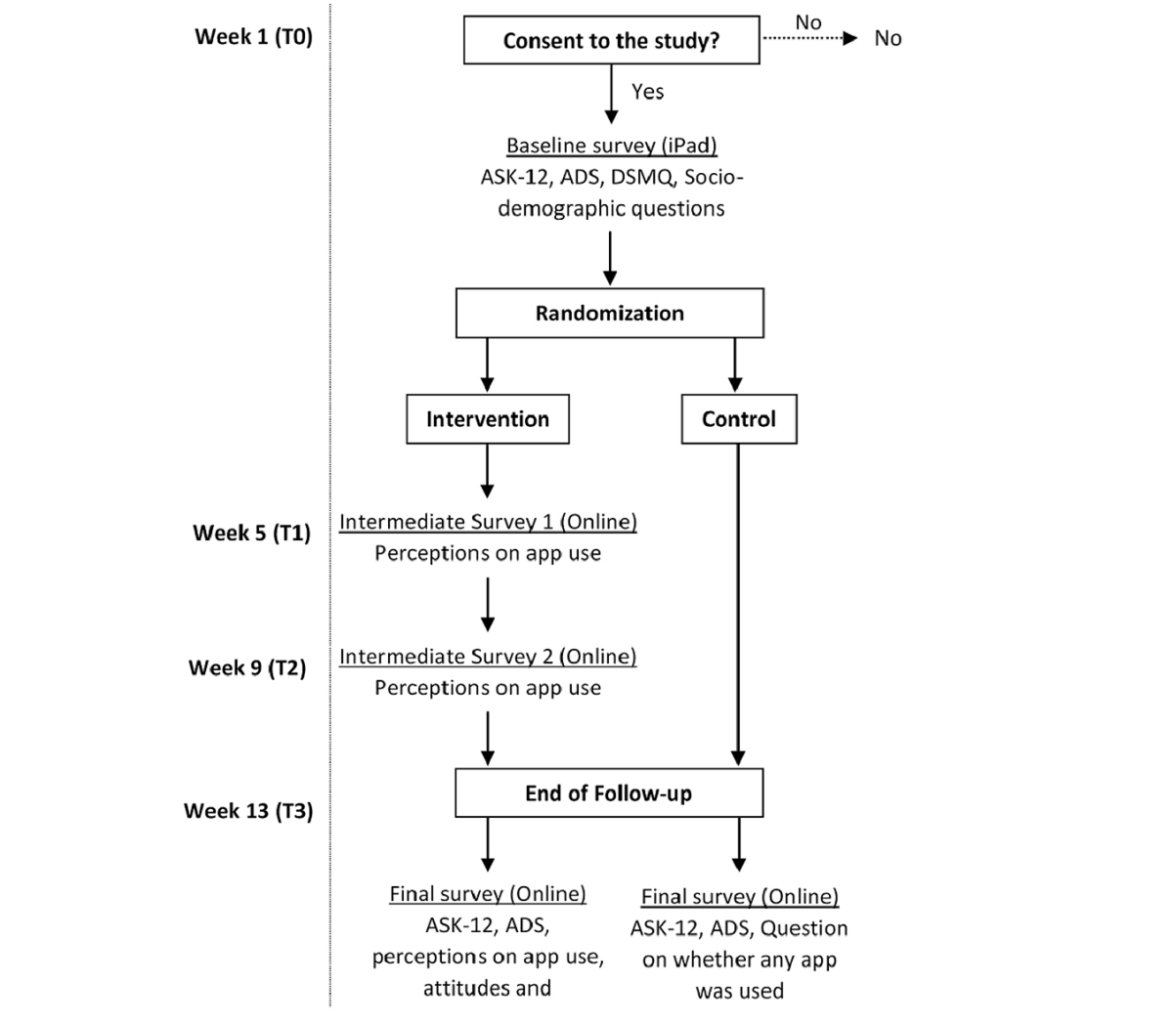
Schedule of outcome measurements. ASK-12: Adherence Starts with Knowledge-12, ADS: Appraisal of Diabetes Scale, DSMQ: Diabetes Self-Management Questionnaire.

Primary outcomes were the feasibility, effectiveness, and acceptability of using a smartphone app to improve medication adherence in a multiethnic Asian population with type 2 diabetes (Figure 1). Feasibility was determined from the recruitment/enrolment rate (percentage of people who reject the study, ie, the number of patients who consented to the study divided by the total number of clinic sessions). Another measure of feasibility is adherence to trial participation, which was assessed by observing intervention group participants’ interaction with the app throughout the intervention through the “Medfriend” feature of the app. The research team, as a “Medfriend,” did not interact with participants during the follow-up period. Reports on the medication-taking status of participants were generated at the end of the intervention (T3) through the app.

Effectiveness was measured with self-reported barriers to medication adherence and assessed at baseline (T0) and poststudy (T3) in both groups by using the ASK-12 questionnaire [30]. The Appraisal of Diabetes Scale (ADS) [34] was administered concurrently with ASK-12 to account for changes (if any) in self-appraisal of diabetes. Acceptability of app use intervention was determined by self-reported perceptions, attitudes, and satisfaction in using the app. Perception toward medication adherence and app usage were assessed at all three time points (T1, T1, and T3), while attitude and satisfaction were only assessed poststudy (T3). Control group participants were asked for the app they used to manage their medication(s) (if any) in the past 3 months in order to assess the level of contamination in the control arm.

Secondary outcomes were diabetes-related health outcomes. Data for assessing secondary outcomes such as anthropometric measures, blood glucose level, and lipid measurements were extracted from clinical records. The following data were also collected for participant profiling and baseline adjustments: data on medications and history of diabetes-related complications from clinical records; sociodemographic data; and responses from a 16-item Diabetes Self-Management Questionnaire (DSMQ) [35] collected at baseline (T0).

### Sample Size

A minimum of 12 participants per treatment arm is necessary to assess the objectives of the study in a two-arm trial [36], and 25 participants per arm is sufficient to account for a dropout rate of about 40% [37]. Therefore, we aimed to recruit and randomize a minimum of 25 participants per arm in 10 weeks of recruitment.

### Randomization

Block randomization (blocks of four) was conducted to ensure a balanced allocation, since we could not anticipate the final sample size. Eligible participants were asked to draw a card from a box with two “intervention” and two “control” cards, which were reset after all four cards were drawn.

### Blinding

The clinical care team was blinded from the study. Participants were only partially blinded, as we had to explain the purpose of the study before randomization. The name of the app was not revealed to participants unless they were randomized into the intervention group or screened out of the study.

### Data Analyses

The intention-to-treat approach was used to analyze the data. We excluded participants who did not complete the final survey due to the lack of poststudy data for pre-post comparison. Intervention group participants who stopped using the app during the study and control group participants who used an app to manage their medications during the study follow-up period were included in the analysis. Scores for the ASK-12, ADS, and DSMQ surveys were computed in accordance to the method suggested by the original authors [30,34,35]. Descriptive analyses were used for baseline comparisons, and linear regressions, controlled for baseline imbalances, were used to compare the pretest and posttest change scores. All statistical assumptions were checked to ensure the accuracy of analyses. Statistical significance was set at *P*<.05. SPSS (version 22; IBM Corp, Armonk, NY) was used for all statistical analyses.

### Ethical Considerations

This study was approved by the SingHealth Centralised Institutional Review Board (Reference: 2018/2563) and the Nanyang Technological University Institutional Review Board (Reference: IRB-2018-09-029) in Singapore. Licenses and permission to use published questionnaires were obtained from the original authors and relevant institutions prior to data collection. We did not prospectively register the trial, as this was a feasibility study.

## Results

### Recruitment

A total of 176 patients were referred and approached for recruitment over 48 three-hour clinic sessions. Overall, 15 patients (8.5%) rejected study participation, which yielded an enrolment rate of approximately 3 (161/48) patients per clinic session. Reasons for rejecting study participation included concerns over the collection of personal data, pressed for time, and refusal to complete the baseline survey. Of the 161 enrolled participants, 110 were not eligible for randomization: 82 (50.9%) self-reported that they were adherent to their medications; 18 (11.2%) were not familiar with smartphone use; 7 (4.3%) refused participation, did not have an email address, or were not confident with completing the online surveys; 2 (1.2%) were already using a smartphone app to complement diabetes management; and 1 (0.6%) could not install the app.

**Figure 2:**
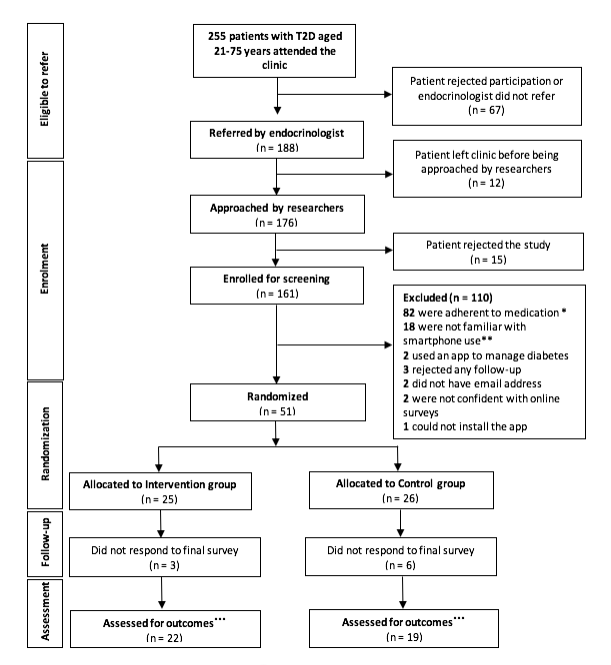
Diagram of participant flow. T2D: type 2 diabetes. *Participants were considered adherent if they answered “disagree/neutral” to the question, “I forget to take my medicines some of the time” or any option within 3 months to the question, “Have you taken a medicine more or less often than prescribed?” in the Adherence Starts with Knowledge-12 questionnaire. **Patients who were not confident of using a new app. ***Three intervention group participants stopped using the app; two control group participants started using an app to manage diabetes during the follow-up period.

A total of 51 (31.7%) participants met the inclusion criteria and were randomized to the intervention (n=25) or control (n=26) group, of which 22 (88.0%) and 19 (73.1%) in the intervention and control group, respectively, completed the postintervention survey (Figure 2). Three intervention group participants (3/22) indicated that they stopped using the app, and two control group participants (2/19) indicated that they used a diabetes self-management app during the follow-up period.

### Randomization

The baseline characteristics of patients included in the analysis are shown in Table 1. Randomization was successful, as there are no statistically significant differences at baseline between groups for sociodemographic and clinical characteristics (eg, blood test results, diabetes-related complications, and anthropometric measurements) and baseline questionnaires (eg, DSMQ and ADS), apart from the number of years with diabetes and the pretest total ASK-12 score. Control group participants lived on an average of 7 years more with diabetes (*P*=.005) and had a lower total ASK-12 score (intervention group: 28.6; control group: 25.5; *P*=.044) compared with the intervention group. Higher ASK-12 scores represent higher barriers to medication adherence.

**Table 1:** Baseline characteristics of patients included in the analyses.

Characteristics			Intervention group (n=22)	Control group (n=19)	*P* value
**Sociodemographic characteristics**					
	Age, median (min-max)		51.5 (22-69)	52 (28-67)	.85^a^
	**Sex, n (%)**				.28
		Male	9 (40.9)	11 (57.9)	
		Female	13 (59.1)	8 (42.1)	
	**Ethnicity, n (%)**				.26
		Chinese	10 (45.5)	12 (63.2)	
		Non-Chinese	12 (54.5)	7 (36.8)	
	**Highest education, n (%)**				.49
		Secondary school and below	11 (50.0)	6 (31.6)	
		Junior college/diploma	4 (18.2)	5 (26.3)	
		University	7 (31.8)	8 (42.1)	
	**Housing (number of rooms), n (%)**				.52
		≤3	2 (9.1)	4 (21.1)	
		4-5	12 (54.5)	10 (52.6)	
		≥5	8 (36.4)	5 (26.3)	
	**Household income (US $), n (%)**				.17
		<4000	6 (30.0)	9 (47.4)	
		4000-6999	4 (20.0)	6 (31.6)	
		≥7000	10 (50.0)	4 (21.1)	
**Clinical characteristics**					
	Number of years with diabetes, median (SD)		11.1 (7.1)	18.3 (8.4)	.005^b^
	Number of different types of medications, median (min-max)		4 (1-9)	4 (1-13)	.47^a^
	**Type of medications, n (%)**				
		Insulin	7 (31.8)	9 (47.4)	.31
		Antihypertensive medication	11 (50.0)	5 (26.3)	.12
		Cholesterol-lowering medication	8 (36.4)	5 (26.3)	.49
	**Medication intensity, n (%)**				
		Oral medications only	7 (31.8)	9 (47.4%)	.051
		Insulin only	0 (0.0)	3 (15.8%)	
		Oral and insulin	15 (68.2)	7 (36.8%)	
	**Anthropometric data, median (min-max)**				
		Body mass index	28.7 (20.2-49.2)	28.3 (21.1-35.6)	.66^b^
	**Diabetes-related complications, n (%)**				
		Proliferative diabetic retinopathy	4 (18.2)	3 (15.8)	>.99^c^
		Peripheral vascular disease	2 (9.1)	3 (15.8)	.65^c^
		Chronic kidney disease (≥stage 3)	3 (13.6)	4 (21.1)	.70^c^
		History of major cardiovascular events	5 (22.7)	3 (15.8)	.69^c^
	**Blood glucose level, median (min-max)**				
		Hemoglobin A_1c_ (%), preintervention	8.2 (5.9-14.8)	8.5 (6.4-11.8)	.57^a^
	**Lipid profile, median (min-max)**				
		Low-density lipoprotein (mmol/L)	2.7 (2.0-6.6)	2.4 (1.3-4.3)	.30^a^
		High-density lipoprotein (mmol/L)	1.1 (0.9-1.7)	1.0 (0.7-2.0)	.09^a^
		Total cholesterol (mmol/L)	4.1 (3.2-8.2)	4.1 (2.5-6.9)	.56^a^
**Baseline questionnaires**					
	**Appraisal of Diabetes Scale, mean (SD)^d^**				
		Total score (baseline)	19.7 (3.7)	19.0 (3.8)	.57
	**Diabetes Self-Management Scale score, mean (SD)^e^**				
		Total score (baseline)	2.0 (0.4)	2.0 (0.3)	.69
	**Adherence Starts with Knowledge-12 medication adherence barrier survey, median (SD)^f^**				
		Total score (baseline)	28.6 (5.2)	25.5 (4.4)	.04^b^

^a^*P*<.05.

^b^Mann-Whitney *U* test for continuous variables.

^c^Fisher exact test for categorical variables with small sample sizes.

^d^Scores (min=7, max=35) are summed up (questions 2 and 6 are reverse scored). Lower scores signify more positive appraisal of diabetes.

^e^Scale scores are computed (min=0, max=4), as there are responses that cannot be scored (eg, “Not part of my treatment”). Items 5, 7, 10, 11, 12, 13, 14, 15, and 16 are reverse scored. Scale scores can be computed as Total_Sum(All)/(16-missing). Higher scores signify better diabetes self-management.

^f^Scores are summed up with reverse scoring for Inconvenience (questions 1-3) and Behavior (questions 8-12). Higher scores signify higher barriers to adherence.

### Outcomes

The mean ASK-12 (adherence barrier) score decreased in the intervention group but increased in the control group. Higher ASK-12 scores signify higher barriers to medication adherence. After baseline adjustment with “years with diabetes” and “baseline ASK-12 score,” the ASK-12 pre-post “change score” was statistically significant (*P*=.01), with the intervention group having a 4.7-point (1.2-8.2) lower mean score than the control group (Table 2).

There were no statistically significant mean differences between groups for baseline-adjusted regression in ADS score, HbA_1c_, lipids, and body mass index (Table 2). Although the mean HbA_1c_ level increased slightly in both groups, the intervention group participants had an average of 0.5% lower increment compared with the control group.

**Table 2:** Adjusted mean differences between treatment groups.

Outcome measure			Intervention				Control				Adjusted mean difference (95% CI)^a^			*P* value		
			Baseline		Poststudy		Baseline		Poststudy							
**Self-reported questionnaires, mean (SD)**																
	Number of participants			22		22		19		19			N/A^b^			N/A
	Adherence Starts with Knowledge-12 scale score^c^			28.6 (5.2)		27.2 (5.8)		25.5 (4.4)		28.5 (7.0)			–4.73 (–8.26 to –1.21)			.01^d^
	Appraisal of Diabetes scale score^e^			19.7 (3.7)		19.4 (3.5)		19.0 (3.8)		19.4 (4.3)^f^			–0.48 (–1.82 to 2.78)			.43^g^
**Clinical measurements**																
	**Blood glucose level**															
		Number of participants		22		19		19		15			N/A			N/A
		Hemoglobin A_1c_ (%)		8.7 (2.4)		9.0 (1.6)		8.6 (1.5)		9.4 (2.4)			–0.42 (–1.89 to 1.06)			.57^g^
	**Lipids**															
		Number of participants		21		17		19		12			N/A			N/A
		Low-density lipoprotein (mmol/L), mean (SD)		3.1 (1.2)		3.1 (0.7)		2.7 (1.0)		2.7 (0.8)			0.11 (–0.20 to 0.06)			.75^g^
		High-density lipoprotein (mmol/L), mean (SD)		1.2 (0.3)		1.2 (0.3)		1.1 (0.3)		1.2 (0.3)			–0.09 (–0.56 to 0.77)			.14
		Total cholesterol (mmol/L), mean (SD)		4.5 (1.2)		4.6 (0.8)		4.2 (1.0)		4.1 (1.1)			–0.02 (–0.69 to 0.72)			.052^g^
	**Anthropometric data**															
		Number of participants		22		18		19		13			N/A			N/A
		Body mass index (kg/m^2^), mean (SD)		29.4 (7.3)		25.2 (12.5)		28.0 (4.0)		27.5 (4.2)			0.02 (–1.13, 1.10)			.98^g^

^a^Adjusted variables for linear regressions: mean baseline ASK-12 score, years with diabetes, baseline of outcome variable.

^b^N/A: not applicable.

^c^Scores are summed up with reverse scoring for Inconvenience (questions 1-3) and Behavior (questions 8-12). Higher scores signify higher barriers to adherence.

^d^*P*<.05

^e^Scores (min=7, max=35) are summed up (questions 2 and 6 are reverse scored). Lower scores signify more positive appraisal of diabetes.

^f^One missing value, n=18.

^g^Normality assumption is violated due to a small number of outliers and small sample sizes per group.

### Adherence to Trial Participation

Three intervention group participants did not complete the final survey, of which, two had intermittent app usage, while one did not use the app from the start. Three other participants who completed the final survey indicated that they stopped using the app between 2 weeks and 2 months into the study, as they did not find the app useful or found it distracting. Two participants who indicated that they were still using the app at the end of the study did not have their medication-taking status tracked, as they were unfamiliar with the app-based medication-logging process. The average individual 12-week medication adherence rate tracked by the app was 38.3%-100% for the remaining 17 participants. Eight participants had 100% adherence for the first 2 weeks of the intervention, which was decreased to four participants by the third week of the intervention.

**Figure 3:**
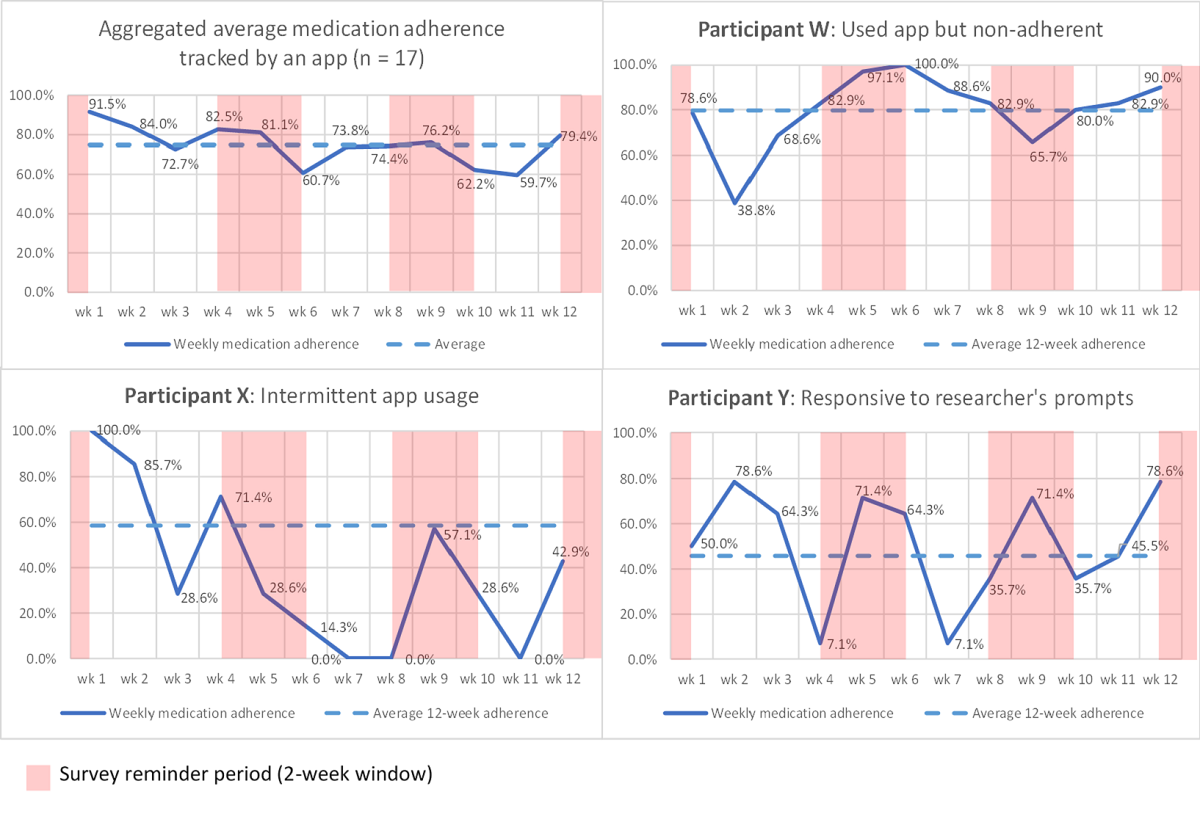
Weekly medication adherence over 12 weeks, extracted from participants’ “Medisafe” reports.

The medication adherence rates tracked by the app also reflect the app usage patterns of the participant. Despite differences in app usage patterns between participants, the aggregated weekly medication adherence tracked by the app did not fall below 50% over the 12 weeks (Figure 3). The graphs in Figure 3 show actual examples of one aggregated and three typical app usage patterns observed in the participants. Medication adherence and health outcomes improved for Participant W who was still occasionally nonadherent to the medication but highly adherent to app usage. Participant X had waning app usage, as perception of the app became less positive over time. Medication adherence and health outcomes did not improve, as participant X ran out of medication in week 7. Several participants exhibited similar cyclical app usage behavior to Participant Y where medication adherence increases when they receive emailed survey reminders. This cyclical pattern was also observed in the aggregated weekly medication adherence tracked by the app.

### Acceptability of the Medication Management Smartphone App

The perception, attitude, and satisfaction of app use (Table 3) show the acceptability of a smartphone app in supporting medication management in the feasibility trial. These surveys were related to participants’ experiences in app use and therefore only administered to the intervention group.

**Table 3:** Perception, attitude, and satisfaction of app use in the intervention group.

Survey topic		Value
**Perception of app usage^a,b^** **, n (%)**		
	Made you more aware of your adherence to medication (Agree; n=21)	19 (90.5)
	Made you more adherent to your medication (Agree; n=21)	17 (81.0)
	Made you more confident in managing your medication (Agree; n=21)	17 (81.0)
	Reduces the stress in managing your medication (Agree; n=21)	14 (66.7)
	Is easy to use (Agree; n=22)	20 (90.9)
	Annoys you when the notification goes off (Agree/neutral; n=20)	16 (80.0)
**Attitude toward app use, n (%)**		
	Would you recommend Medisafe to another person with the same condition? (Yes)	21 (95.5)
	Would you trust your doctor to recommend an app for you to manage your condition? (Yes)	21 (95.5)
	Will you continue to use the Medisafe app after today? (Yes)	19 (86.4)
**Satisfaction, median (min-max)**		
	On a scale of 1 to 10, with 10 being very satisfied, how would you rate your experience in using an app for managing your medication?	8 (1-10)

^a^There is a “Not applicable” option for “Perception on app usage” questions, which caused the denominator to differ.

^b^In response to the question, “Thinking about the past few days, how far do you agree that the app?”.

#### Perception of App Usage

The perception of app usage was generally positive among respondents, with the majority (>80%) agreeing that the app made them more aware of the importance of medication adherence, more confident in managing their medication, and more adherent to their medication. For 90.9% of the respondents, the app was easy to use. However, use of the app did not reduce medication management stress in 34% of the respondents, and 80% of the respondents found the reminder notification annoying.

#### Attitude Toward App Use

The attitude toward app use was generally positive, with 95.5% of the respondents answering “Yes” to recommending the app to another person with the same condition and trusting their doctor to recommend an app for them to manage type 2 diabetes. The majority of respondents (86.4%) indicated they would continue to use the app after the study.

#### Satisfaction

General satisfaction was high, with a median score of 8 on a scale of 1-10. Participants who stopped using the app provided lower scores. For example, one participant who stopped using the app provided a score of 1/10.

#### Participant Feedback

Two participants would have liked to add their spouses as a “Medfriend” but could not do so, as the free version only allowed the addition of one “Medfriend” (ie, the study team). Other feedback include suggestions to incorporate the doctor’s appointment scheduling and other diabetes self-management features, simplifying the app interface, educating participants on manipulating the settings, and integrating some of the hospital’s services with the app. Although Medisafe is a third-party app, patients would prefer integrating all health services into a one-stop reliable and personalized platform.

## Discussion

### Principal Findings

We established the feasibility of using a smartphone app to improve medication adherence in patients with type 2 diabetes managed at a public diabetes specialist outpatient center in Singapore through a pilot study. The medication nonadherence rate determined by the study (49.1%) falls within the range of rates reported by other studies in Singapore and internationally using a variety of measurement tools [6,38]. We observed significantly lower self-reported barriers to medication adherence in the intervention group compared with the control group but no improvement in the HbA_1c_ level. This concurs with the findings of a similar US study, which observed improvement in self-reported medication adherence but no change in blood pressure over 12 weeks [18].

The control group had slightly lower HbA_1c_ level, barriers to medication adherence, and more positive appraisal of diabetes at baseline compared with the intervention group. This observation was reversed 12 weeks later when the intervention group had slightly better outcomes in all three measurements. Improvement in barriers to medication adherence in the intervention group is likely attributed to medication-taking reinforcements by the app and monthly email reminders to complete the online surveys. Adherence reinforcements will likely lead to short-term improvement in medication adherence [39].

We observed increased HbA_1c_ levels in both groups, which is attributed to the follow-up period falling within a few holiday seasons (ie, Diwali, Christmas, and Chinese New Year) where festive feasting in Asian cultures (ie, Singapore) is likely [40]. A different intervention period may change the study outcomes, although we acknowledge that the degree of medication nonadherence, personal motivation, and response to treatment can affect the HbA_1c_ levels and add complexity to the interpretation of outcomes [41].

We observed various factors that influenced study feasibility. First, physician advocacy is important in encouraging the uptake of new health interventions. The majority (>85%) of patients referred by their endocrinologists were willing to provide informed consent and complete the baseline questionnaire. Most of the intervention group participants also indicated that they would trust their doctor to recommend an app to manage their condition. Second, the use of digital data collection tools (ie, REDCap) minimized data entry errors and human resources required for data collection.

Third, participants’ digital literacy and the app’s usability influence adherence to the intervention and satisfaction. Many older participants have difficulty adjusting the app settings, which caused the reminders to become a distraction instead. Fourth, reasons for medication nonadherence affect study feasibility and outcomes. For people with polypharmacy, an app may help to better organize medication-taking schedules. However, this does not solve barriers to medication adherence such as the inconvenience of taking multiple medications, medication side effects, or fear of injections. Lastly, the health-seeking behavior of participants will influence the study outcomes. For example, one motivated participant in the control group started using an app for diabetes management during study follow-up and achieved >0.5% HbA_1c_ improvement in 12 weeks.

There were limitations to the study. We were unable to observe app usage patterns of a few participants who changed smartphones during study follow-up. Medication adherence rates in the control group were also not tracked for comparison. Self-reported tools are subjective to a patient’s own judgement and social desirability bias; hence, actual medication adherence may not be accurately reflected. We observed patients who over- and underreported their medication adherence status and problems with survey interpretation. For example, when the researchers verbally asked (at baseline), “How likely do you think your diabetes will worsen in the next few years?” a few participants answered “I hope it will not worsen” instead of choosing a Likert scale response. The study may not be generalizable to all people with diabetes, as tertiary specialist outpatient clinics are likely to manage more complex cases that cannot be managed in the primary care setting. Lastly, contamination may have occurred when the control group participants were exposed to the idea of using an app for type 2 diabetes medication management.

This study allowed us to better understand the impact of a health app on patients with type 2 diabetes and identify potential problems that could occur before scaling up the study. One registered trial using a self-developed smartphone app to improve the 6-month medication adherence among patients with type 2 diabetes in Singapore was withdrawn due to poor patient recruitment [42]. Therefore, we conducted a pilot trial with a commercial app to first evaluate factors that are important for implementing a full trial. Our findings suggest that should a full randomized controlled trial be conducted, a five-fold scale-up is required to achieve full trial power under the same conditions. This can be achieved with the involvement of more physicians, more study sites, or a longer recruitment period. Future studies should assess factors that could enhance the usability of apps in older adults who are less technologically savvy. The app usage behavior of different patient subgroups and interaction between various diabetes app features can also be explored.

### Conclusions

Our feasibility study found that a smartphone app intervention for medication nonadherent patients with type 2 diabetes in a developed Asian setting is feasible and acceptable, improved awareness of medication adherence, and reduced self-reported barriers to medication adherence. Digital literacy, health-seeking behavior, app usability, and the time period of the intervention are factors that influenced feasibility.

## Appendix

Multimedia Appendix 1 CONSORT‐EHEALTH checklist (V 1.6.1).

## Abbreviations

ASK-12: Adherence Starts with Knowledge-12
ADS: Appraisal of Diabetes Scale
CONSORT: Consolidated Standards of Reporting Trials
DSMQ: Diabetes Self-Management Questionnaire
HbA_1c_: hemoglobin A_1c_
mERA: mobile health evidence reporting and assessment
REDCap: Research Electronic Data Capture
