# Correction: A Smartphone App to Improve Medication Adherence in Patients With Type 2 Diabetes in Asia: Feasibility Randomized Controlled Trial

**DOI:** 10.2196/18411

**Published:** 2020-04-29

**Authors:** Zhilian Huang, Eberta Tan, Elaine Lum, Peter Sloot, Bernhard Otto Boehm, Josip Car

**Affiliations:** 1 Centre for Population Health Sciences Lee Kong Chian School of Medicine Nanyang Technological University Singapore Singapore; 2 NTU Institute for Health Technologies Interdisciplinary Graduate School Nanyang Technological University Singapore Singapore; 3 Department of Endocrinology Changi General Hospital Singapore Singapore; 4 Institute of Health and Biomedical Innovation Queensland University of Technology Queensland Australia; 5 School of Clinical Sciences Faculty of Health Queensland University of Technology Queensland Australia; 6 Complexity Institute Nanyang Technological University Singapore Singapore; 7 Institute for Advanced Science University of Amsterdam Amsterdam Netherlands; 8 ITMO University Saint Petersburg Russian Federation; 9 Lee Kong Chian School of Medicine Nanyang Technological University Singapore Singapore; 10 Department of Endocrinology Tan Tock Seng Hospital Singapore Singapore

The authors of “A Smartphone App to Improve Medication Adherence in Patients With Type 2 Diabetes in Asia: Feasibility Randomized Controlled Trial” (JMIR Mhealth Uhealth 2019;7(9):e14914) noticed an error in the Results section of their published article. Under the “Adherence to Trial Participation” subsection of the Results section, the following sentence:

Eight participants had 100% adherence for the first 2 weeks of the intervention, which was decreased to 4% by the third week of the intervention.

Has been changed to:

Eight participants had 100% adherence for the first 2 weeks of the intervention, which was decreased to four participants by the third week of the intervention.

This change does not affect any of the data presented in the Results section of the paper.

The correction will appear in the online version of the paper on the JMIR website on April 29, 2020, together with the publication of this correction notice. Because this was made after submission to PubMed, PubMed Central, and other full-text repositories, the corrected article has also been resubmitted to those repositories.

