# Supplementary material for: A Smartphone App to Improve Medication Adherence in Patients With Type 2 Diabetes in Asia: Feasibility Randomized Controlled Trial
Source: JMIR Mhealth Uhealth. 2019 Sep 12;7(9):e14914. doi: 10.2196/14914 (PMC6746066; doi:10.2196/14914)
Supplement: Multimedia Appendix 1 [file mhealth_v7i9e14914_app1.pdf]

# CONSORT-EHEALTH (V 1.6.1) - Submission/Publication Form

The CONSORT-EHEALTH checklist is intended for authors of randomized trials evaluating web-based and Internet-based applications/interventions, including mobile interventions, electronic games (incl multiplayer games), social media, certain telehealth applications, and other interactive and/or networked electronic applications. Some of the items (e.g. all subitems under item 5 - description of the intervention) may also be applicable for other study designs.

The goal of the CONSORT EHEALTH checklist and guideline is to be
a) a guide for reporting for authors of RCTs,
b) to form a basis for appraisal of an ehealth trial (in terms of validity)

CONSORT-EHEALTH items/subitems are MANDATORY reporting items for studies published in the Journal of Medical Internet Research and other journals / scientific societies endorsing the checklist.

Items numbered 1., 2., 3., 4a., 4b etc are original CONSORT or CONSORT-NPT (non-pharmacologic treatment) items.
Items with Roman numerals (i., ii, iii, iv etc.) are CONSORT-EHEALTH extensions/clarifications.

As the CONSORT-EHEALTH checklist is still considered in a formative stage, we would ask that you also RATE ON A SCALE OF 1-5 how important/useful you feel each item is FOR THE PURPOSE OF THE CHECKLIST and reporting guideline (optional).

Mandatory reporting items are marked with a red *.
In the textboxes, either copy & paste the relevant sections from your manuscript into this form - please include any quotes from your manuscript in QUOTATION MARKS,
or answer directly by providing additional information not in the manuscript, or elaborating on why the item was not relevant for this study.

YOUR ANSWERS WILL BE PUBLISHED AS  A SUPPLEMENTARY FILE TO YOUR PUBLICATION IN JMIR AND ARE CONSIDERED PART OF YOUR PUBLICATION (IF ACCEPTED).
Please fill in these questions diligently. Information will not be copyedited, so please use proper spelling and grammar, use correct capitalization, and avoid abbreviations.

DO NOT FORGET TO SAVE AS PDF _AND_ CLICK THE SUBMIT BUTTON SO YOUR ANSWERS ARE IN OUR DATABASE !!!

Citation Suggestion (if you append the pdf as Appendix we suggest to cite this paper in the caption):
Eysenbach G, CONSORT-EHEALTH Group
CONSORT-EHEALTH: Improving and Standardizing Evaluation Reports of Web-based and Mobile Health Interventions
J Med Internet Res 2011;13(4):e126
URL: http://www.jmir.org/2011/4/e126/
doi: 10.2196/jmir.1923
PMID: 22209829

* Required

## Your name *
First Last

Zhilian Huang

## Primary Affiliation (short), City, Country *
University of Toronto, Toronto, Canada

Nanyang Technological University, Sir

## Your e-mail address *
abc@gmail.com

ZHUANG014@e.ntu.edu.sg

## Title of your manuscript *
Provide the (draft) title of your manuscript.

A Smartphone App to Improve Medication Adherence in Patients With Type 2 Diabetes in Asia: Feasibility Randomized Controlled Trial

## Name of your App/Software/Intervention *

If there is a short and a long/alternate name, write the short name first and add the long name in brackets.

Medisafe app

## Evaluated Version (if any)

e.g. "V1", "Release 2017-03-01", "Version 2.0.27913"

Your answer

## Language(s) *

What language is the intervention/app in? If multiple languages are available, separate by comma (e.g. "English, French")

English

## URL of your Intervention Website or App

e.g. a direct link to the mobile app on app in appstore (itunes, Google Play), or URL of the website. If the intervention is a DVD or hardware, you can also link to an Amazon page.

https://www.medisafeapp.com/

## URL of an image/screenshot (optional)

Your answer

## Accessibility *

Can an enduser access the intervention presently?

- (selected) access is free and open
- access only for special usergroups, not open
- access is open to everyone, but requires payment/subscription/in-app purchases
- app/intervention no longer accessible
- Other:

## Primary Medical Indication/Disease/Condition *

e.g. "Stress", "Diabetes", or define the target group in brackets after the condition, e.g. "Autism (Parents of children with)", "Alzheimers (Informal Caregivers of)"

Type 2 diabetes

## Primary Outcomes measured in trial *

comma-separated list of primary outcomes reported in the trial

feasibility (Recruitment/enrolment ra[illegible]

## Secondary/other outcomes

Are there any other outcomes the intervention is expected to affect?

BMI, HbA1c, HDL, LDL, Total cholesterol

## Recommended "Dose" *

What do the instructions for users say on how often the app should be used?

- (●) Approximately Daily
- ( ) Approximately Weekly
- ( ) Approximately Monthly
- ( ) Approximately Yearly
- ( ) "as needed"
- ( ) Other:

Approx. Percentage of Users (starters) still using the app as recommended after 3 months *

- ○ unknown / not evaluated
- ○ 0-10%
- ○ 11-20%
- ○ 21-30%
- ○ 31-40%
- ○ 41-50%
- ○ 51-60%
- ○ 61-70%
- ◉ 71%-80%
- ○ 81-90%
- ○ 91-100%
- ○ Other:

## Overall, was the app/intervention effective? *

- ( ) yes: all primary outcomes were significantly better in intervention group vs control
- (•) partly: SOME primary outcomes were significantly better in intervention group vs control
- ( ) no statistically significant difference between control and intervention
- ( ) potentially harmful: control was significantly better than intervention in one or more outcomes
- ( ) inconclusive: more research is needed
- ( ) Other:

## Article Preparation Status/Stage *

At which stage in your article preparation are you currently (at the time you fill in this form)

- ( ) not submitted yet - in early draft status
- ( ) not submitted yet - in late draft status, just before submission
- ( ) submitted to a journal but not reviewed yet
- ( ) submitted to a journal and after receiving initial reviewer comments
- (•) submitted to a journal and accepted, but not published yet
- ( ) published
- ( ) Other:

## Journal *

If you already know where you will submit this paper (or if it is already submitted), please provide the journal name (if it is not JMIR, provide the journal name under "other")

- ○ not submitted yet / unclear where I will submit this
- ○ Journal of Medical Internet Research (JMIR)
- ◉ JMIR mHealth and UHealth
- ○ JMIR Serious Games
- ○ JMIR Mental Health
- ○ JMIR Public Health
- ○ JMIR Formative Research
- ○ Other JMIR sister journal
- ○ Other:

## Is this a full powered effectiveness trial or a pilot/feasibility trial? *

- ◉ Pilot/feasibility
- ○ Fully powered

## Manuscript tracking number *

If this is a JMIR submission, please provide the manuscript tracking number under "other" (The ms tracking number can be found in the submission acknowledgement email, or when you login as author in JMIR. If the paper is already published in JMIR, then the ms tracking number is the four-digit number at the end of the DOI, to be found at the bottom of each published article in JMIR)

- ○ no ms number (yet) / not (yet) submitted to / published in JMIR
- ◉ Other: 14914

# TITLE AND ABSTRACT

## 1a) TITLE: Identification as a randomized trial in the title

## 1a) Does your paper address CONSORT item 1a? *

I.e does the title contain the phrase "Randomized Controlled Trial"? (if not, explain the reason under "other")

- ◉ yes
- ○ Other:

## 1a-i) Identify the mode of delivery in the title

Identify the mode of delivery. Preferably use “web-based” and/or “mobile” and/or “electronic game” in the title. Avoid ambiguous terms like “online”, “virtual”, “interactive”. Use “Internet-based” only if Intervention includes non-web-based Internet components (e.g. email), use “computer-based” or “electronic” only if offline products are used. Use “virtual” only in the context of “virtual reality” (3-D worlds). Use “online” only in the context of “online support groups”. Complement or substitute product names with broader terms for the class of products (such as “mobile” or “smart phone” instead of “iphone”), especially if the application runs on different platforms.

| | 1 | 2 | 3 | 4 | 5 | |
|---|---|---|---|---|---|---|
| subitem not at all important | ○ | ○ | ○ | ○ | ◉ | essential |

## Does your paper address subitem 1a-i? *

Copy and paste relevant sections from manuscript title (include quotes in quotation marks "like this" to indicate direct quotes from your manuscript), or elaborate on this item by providing additional information not in the ms, or briefly explain why the item is not applicable/relevant for your study

Yes. The mode of delivery: "a smartphone app" was included in the title

## 1a-ii) Non-web-based components or important co-interventions in title

Mention non-web-based components or important co-interventions in title, if any (e.g., "with telephone support").

| | 1 | 2 | 3 | 4 | 5 | |
|---|---|---|---|---|---|---|
| subitem not at all important | ○ | ○ | ● | ○ | ○ | essential |

## Does your paper address subitem 1a-ii?

Copy and paste relevant sections from manuscript title (include quotes in quotation marks "like this" to indicate direct quotes from your manuscript), or elaborate on this item by providing additional information not in the ms, or briefly explain why the item is not applicable/relevant for your study

No. There are no co-interventions in the title of the manuscript

## 1a-iii) Primary condition or target group in the title

Mention primary condition or target group in the title, if any (e.g., "for children with Type I Diabetes") Example: A Web-based and Mobile Intervention with Telephone Support for Children with Type I Diabetes: Randomized Controlled Trial

| | 1 | 2 | 3 | 4 | 5 | |
|---|---|---|---|---|---|---|
| subitem not at all important | ○ | ○ | ○ | ○ | ● | essential |

## Does your paper address subitem 1a-iii? *

Copy and paste relevant sections from manuscript title (include quotes in quotation marks "like this" to indicate direct quotes from your manuscript), or elaborate on this item by providing additional information not in the ms, or briefly explain why the item is not applicable/relevant for your study

Yes. The focus of this study is on people with type 2 diabetes as described in the title.

# 1b) ABSTRACT: Structured summary of trial design, methods, results, and conclusions

NPT extension: Description of experimental treatment, comparator, care providers, centers, and blinding status.

## 1b-i) Key features/functionalities/components of the intervention and comparator in the METHODS section of the ABSTRACT

Mention key features/functionalities/components of the intervention and comparator in the abstract. If possible, also mention theories and principles used for designing the site. Keep in mind the needs of systematic reviewers and indexers by including important synonyms. (Note: Only report in the abstract what the main paper is reporting. If this information is missing from the main body of text, consider adding it)

| | 1 | 2 | 3 | 4 | 5 | |
|---|---|---|---|---|---|---|
| subitem not at all important | ○ | ○ | ○ | ○ | ● | essential |

## Does your paper address subitem 1b-i? *

Copy and paste relevant sections from the manuscript abstract (include quotes in quotation marks "like this" to indicate direct quotes from your manuscript), or elaborate on this item by providing additional information not in the ms, or briefly explain why the item is not applicable/relevant for your study

Yes. The comparator "control:usual care; intervention: usual care+Medisafe app" and the components of the intervention "The intervention group received email reminders to complete online surveys monthly, while the control group only received an email reminder(s) at the end of the study. Barriers to medication adherence and self-appraisal of diabetes were assessed using the Adherence Starts with Knowledge-12 (ASK-12) and Appraisal of Diabetes Scale (ADS) questionnaires at baseline and poststudy in both groups. Perception toward medication adherence and app usage, attitude, and satisfaction were assessed in the intervention group during and after the follow-up period. Sociodemographic data were collected at baseline. Clinical data (ie, hemoglobin A1c, body mass index, low-density lipoprotein, high-density lipoprotein, and total cholesterol) were extracted from patients' electronic

## 1b-ii) Level of human involvement in the METHODS section of the ABSTRACT

Clarify the level of human involvement in the abstract, e.g., use phrases like "fully automated" vs. "therapist/nurse/care provider/physician-assisted" (mention number and expertise of providers involved, if any). (Note: Only report in the abstract what the main paper is reporting. If this information is missing from the main body of text, consider adding it)

| | 1 | 2 | 3 | 4 | 5 | |
|---|---|---|---|---|---|---|
| subitem not at all important | ○ | ○ | ○ | ◉ | ○ | essential |

## Does your paper address subitem 1b-ii?

Copy and paste relevant sections from the manuscript abstract (include quotes in quotation marks "like this" to indicate direct quotes from your manuscript), or elaborate on this item by providing additional information not in the ms, or briefly explain why the item is not applicable/relevant for your study

Yes. We specified that "Recruitment occurred at a public tertiary diabetes specialist outpatient center in Singapore. The intervention group received email reminders to complete online surveys monthly, while the control group only received an email reminder(s) at the end of the study."

## 1b-iii) Open vs. closed, web-based (self-assessment) vs. face-to-face assessments in the METHODS section of the ABSTRACT

Mention how participants were recruited (online vs. offline), e.g., from an open access website or from a clinic or a closed online user group (closed usergroup trial), and clarify if this was a purely web-based trial, or there were face-to-face components (as part of the intervention or for assessment). Clearly say if outcomes were self-assessed through questionnaires (as common in web-based trials). Note: In traditional offline trials, an open trial (open-label trial) is a type of clinical trial in which both the researchers and participants know which treatment is being administered. To avoid confusion, use “blinded” or “unblinded” to indicated the level of blinding instead of “open”, as “open” in web-based trials usually refers to “open access” (i.e. participants can self-enrol). (Note: Only report in the abstract what the main paper is reporting. If this information is missing from the main body of text, consider adding it)

| | 1 | 2 | 3 | 4 | 5 | |
|---|---|---|---|---|---|---|
| subitem not at all important | ○ | ○ | ○ | ○ | ● | essential |

## Does your paper address subitem 1b-iii?

Copy and paste relevant sections from the manuscript abstract (include quotes in quotation marks "like this" to indicate direct quotes from your manuscript), or elaborate on this item by providing additional information not in the ms, or briefly explain why the item is not applicable/relevant for your study

Yes. "Recruitment occurred at a public tertiary diabetes specialist outpatient center in Singapore. The intervention group received email reminders to complete online surveys monthly, while the control group only received an email reminder(s) at the end of the study. Barriers to medication adherence and self-appraisal of diabetes were assessed using the Adherence Starts with Knowledge-12 (ASK-12) and Appraisal of Diabetes Scale (ADS) questionnaires at baseline and poststudy in both groups. Perception toward medication adherence and app usage, attitude, and satisfaction were assessed in the intervention group during and after the follow-up period. Sociodemographic data were collected at baseline. Clinical data (ie, hemoglobin A1c, body mass index, low-density lipoprotein, high-density lipoprotein, and total cholesterol) were extracted

## 1b-iv) RESULTS section in abstract must contain use data

Report number of participants enrolled/assessed in each group, the use/uptake of the intervention (e.g., attrition/adherence metrics, use over time, number of logins etc.), in addition to primary/secondary outcomes. (Note: Only report in the abstract what the main paper is reporting. If this information is missing from the main body of text, consider adding it)

| | 1 | 2 | 3 | 4 | 5 | |
|---|---|---|---|---|---|---|
| subitem not at all important | ○ | ○ | ○ | ○ | ● | essential |

## Does your paper address subitem 1b-iv?

Copy and paste relevant sections from the manuscript abstract (include quotes in quotation marks "like this" to indicate direct quotes from your manuscript), or elaborate on this item by providing additional information not in the ms, or briefly explain why the item is not applicable/relevant for your study

Yes. "Fifty-one (intervention group: 25; control group: 26) participants were randomized, of which, 41 (intervention group: 22 [88.0%]; control group: 19 [73.1%]) completed the poststudy survey."

## 1b-v) CONCLUSIONS/DISCUSSION in abstract for negative trials

Conclusions/Discussions in abstract for negative trials: Discuss the primary outcome - if the trial is negative (primary outcome not changed), and the intervention was not used, discuss whether negative results are attributable to lack of uptake and discuss reasons. (Note: Only report in the abstract what the main paper is reporting. If this information is missing from the main body of text, consider adding it)

| | 1 | 2 | 3 | 4 | 5 | |
|---|---|---|---|---|---|---|
| subitem not at all important | ○ | ○ | ○ | ○ | ◉ | essential |

## Does your paper address subitem 1b-v?

Copy and paste relevant sections from the manuscript abstract (include quotes in quotation marks "like this" to indicate direct quotes from your manuscript), or elaborate on this item by providing additional information not in the ms, or briefly explain why the item is not applicable/relevant for your study

Yes. We mentioned that the smartphone app "did not improve clinical outcomes in a developed Asian setting". The reasons attributed to clinical outcomes was discussed in the manuscript but was not mentioned in the abstract as they were secondary outcomes.

## INTRODUCTION

## 2a) In INTRODUCTION: Scientific background and explanation of rationale

## 2a-i) Problem and the type of system/solution

Describe the problem and the type of system/solution that is object of the study: intended as stand-alone intervention vs. incorporated in broader health care program? Intended for a particular patient population? Goals of the intervention, e.g., being more cost-effective to other interventions, replace or complement other solutions? (Note: Details about the intervention are provided in “Methods” under 5)

| | 1 | 2 | 3 | 4 | 5 | |
|---|---|---|---|---|---|---|
| subitem not at all important | ○ | ○ | ○ | ◉ | ○ | essential |

## Does your paper address subitem 2a-i? *

Copy and paste relevant sections from the manuscript (include quotes in quotation marks "like this" to indicate direct quotes from your manuscript), or elaborate on this item by providing additional information not in the ms, or briefly explain why the item is not applicable/relevant for your study

Yes. The problem was "a paucity of studies on the efficacy and implementation of smartphone apps in supporting medication taking" and "diabetes and medication adherence app interventions are not well studied in Asian populations" and the solution was to conduct a randomized feasibility trial among people with type 2 diabetes in settings that are less studied (ie, an Asian setting).

## 2a-ii) Scientific background, rationale: What is known about the (type of) system

Scientific background, rationale: What is known about the (type of) system that is the object of the study (be sure to discuss the use of similar systems for other conditions/diagnoses, if appropiate), motivation for the study, i.e. what are the reasons for and what is the context for this specific study, from which stakeholder viewpoint is the study performed, potential impact of findings [2]. Briefly justify the choice of the comparator.

| | 1 | 2 | 3 | 4 | 5 | |
|---|---|---|---|---|---|---|
| subitem not at all important | ○ | ○ | ○ | ◉ | ○ | essential |

### Does your paper address subitem 2a-ii? *

Copy and paste relevant sections from the manuscript (include quotes in quotation marks "like this" to indicate direct quotes from your manuscript), or elaborate on this item by providing additional information not in the ms, or briefly explain why the item is not applicable/relevant for your study

Yes. The rationale for focusing on Type 2 diabetes, an Asian setting and for conducting a feasibility trial was described in the introduction. For example: "Poor adherence to diabetes medication results in suboptimal glycemic control which increases the risk of diabetes-related complications", "Given the acceleration of mobile connectivity in the Asia Pacific region in recent yearsNo, it is important to investigate the receptivity and usage of apps for diabetes medication management in Asian populations with high mobile penetration", and "In view of the challenges with evaluating complex health interventions, a feasibility and piloting phase to optimize study design and evaluation is warranted".

## 2b) In INTRODUCTION: Specific objectives or hypotheses

### Does your paper address CONSORT subitem 2b? *

Copy and paste relevant sections from the manuscript (include quotes in quotation marks "like this" to indicate direct quotes from your manuscript), or elaborate on this item by providing additional information not in the ms, or briefly explain why the item is not applicable/relevant for your study

Yes. "We aimed to determine the feasibility, effectiveness, acceptability, and clinical outcomes of using a smartphone app to improve medication adherence in a multiethnic Asian population with type 2 diabetes through a pilot study. Our objectives were to assess the recruitment rate, changes in self-reported barriers to medication adherence, diabetes-related health outcomes, app usage behavior, and satisfaction levels."

## METHODS

## 3a) Description of trial design (such as parallel, factorial) including allocation ratio

### Does your paper address CONSORT subitem 3a? *

Copy and paste relevant sections from the manuscript (include quotes in quotation marks "like this" to indicate direct quotes from your manuscript), or elaborate on this item by providing additional information not in the ms, or briefly explain why the item is not applicable/relevant for your study

Yes. "We used a randomized two-arm pre-posttest control group design with a 12-week follow-up period. All participants received usual care, while the intervention group participants additionally downloaded and used the Medisafe app on their personal smartphones during the study."

## 3b) Important changes to methods after trial commencement (such as eligibility criteria), with reasons

### Does your paper address CONSORT subitem 3b? *

Copy and paste relevant sections from the manuscript (include quotes in quotation marks "like this" to indicate direct quotes from your manuscript), or elaborate on this item by providing additional information not in the ms, or briefly explain why the item is not applicable/relevant for your study

No. The smartphone app used in this study is a third party commercial app which we had an agreement to use for research purposes. The research team had no control over bug fixes, but problems with the app were provided as feedback to the company. Medisafe also provided technical support on issues encountered by research team . We did not report these issues as they did not directly affect the participant's app use.

## 3b-i) Bug fixes, Downtimes, Content Changes

Bug fixes, Downtimes, Content Changes: ehealth systems are often dynamic systems. A description of changes to methods therefore also includes important changes made on the intervention or comparator during the trial (e.g., major bug fixes or changes in the functionality or content) (5-iii) and other "unexpected events" that may have influenced study design such as staff changes, system failures/downtimes, etc. [2].

| | 1 | 2 | 3 | 4 | 5 | |
|---|---|---|---|---|---|---|
| subitem not at all important | ○ | ○ | ◉ | ○ | ○ | essential |

## Does your paper address subitem 3b-i?

Copy and paste relevant sections from the manuscript (include quotes in quotation marks "like this" to indicate direct quotes from your manuscript), or elaborate on this item by providing additional information not in the ms, or briefly explain why the item is not applicable/relevant for your study

No. Issues with the Medisafe app did not lead to changes in study methods.

## 4a) Eligibility criteria for participants

## Does your paper address CONSORT subitem 4a? *

Copy and paste relevant sections from the manuscript (include quotes in quotation marks "like this" to indicate direct quotes from your manuscript), or elaborate on this item by providing additional information not in the ms, or briefly explain why the item is not applicable/relevant for your study

Yes

## 4a-i) Computer / Internet literacy

Computer / Internet literacy is often an implicit "de facto" eligibility criterion - this should be explicitly clarified.

| | 1 | 2 | 3 | 4 | 5 | |
|---|---|---|---|---|---|---|
| subitem not at all important | ○ | ○ | ○ | ◉ | ○ | essential |

## Does your paper address subitem 4a-i?

Copy and paste relevant sections from the manuscript (include quotes in quotation marks "like this" to indicate direct quotes from your manuscript), or elaborate on this item by providing additional information not in the ms, or briefly explain why the item is not applicable/relevant for your study

Yes. "secondary inclusion criteria for randomization into the study are self-reported medication nonadherence, digital literacy, and nonuse of a medication management app in the past 2 weeks"

## 4a-ii) Open vs. closed, web-based vs. face-to-face assessments:

Open vs. closed, web-based vs. face-to-face assessments: Mention how participants were recruited (online vs. offline), e.g., from an open access website or from a clinic, and clarify if this was a purely web-based trial, or there were face-to-face components (as part of the intervention or for assessment), i.e., to what degree got the study team to know the participant. In online-only trials, clarify if participants were quasi-anonymous and whether having multiple identities was possible or whether technical or logistical measures (e.g., cookies, email confirmation, phone calls) were used to detect/prevent these.

| | 1 | 2 | 3 | 4 | 5 | |
|---|---|---|---|---|---|---|
| subitem not at all important | ○ | ○ | ○ | ● | ○ | essential |

## Does your paper address subitem 4a-ii? *

Copy and paste relevant sections from the manuscript (include quotes in quotation marks "like this" to indicate direct quotes from your manuscript), or elaborate on this item by providing additional information not in the ms, or briefly explain why the item is not applicable/relevant for your study

Yes. The term "face-to-face" was not mentioned, but the contact participants have with the research team were detailed in the methods section. "Patients with type 2 diabetes attending their scheduled clinic appointments, who met the referral inclusion criteria, were referred to the researchers by their endocrinologist." "Participants were assisted by the researchers to download the Medisafe app on their personal smartphone, set their medication schedule and reminder on the app, and to use the app. Participants were asked to use the app freely outside the health care setting and add the research group as a "Medfriend" for their medication-taking patterns to be observed." "All follow-up surveys were conducted online via a unique link sent to the participants' email address."

## 4a-iii) Information giving during recruitment

Information given during recruitment. Specify how participants were briefed for recruitment and in the informed consent procedures (e.g., publish the informed consent documentation as appendix, see also item X26), as this information may have an effect on user self-selection, user expectation and may also bias results.

| | 1 | 2 | 3 | 4 | 5 | |
|---|---|---|---|---|---|---|
| subitem not at all important | ○ | ○ | ○ | ● | ○ | essential |

## Does your paper address subitem 4a-iii?

Copy and paste relevant sections from the manuscript (include quotes in quotation marks "like this" to indicate direct quotes from your manuscript), or elaborate on this item by providing additional information not in the ms, or briefly explain why the item is not applicable/relevant for your study

Yes. "At the point of consent seeking, researchers explained to potential participants that they may or may not be selected for the study, depending on their eligibility, which can only be determined after they respond to the baseline questionnaire."

## 4b) Settings and locations where the data were collected

### Does your paper address CONSORT subitem 4b? *

Copy and paste relevant sections from the manuscript (include quotes in quotation marks "like this" to indicate direct quotes from your manuscript), or elaborate on this item by providing additional information not in the ms, or briefly explain why the item is not applicable/relevant for your study

Yes. The setting of data collection were clearly described in the manuscript

### 4b-i) Report if outcomes were (self-)assessed through online questionnaires

Clearly report if outcomes were (self-)assessed through online questionnaires (as common in web-based trials) or otherwise.

| | 1 | 2 | 3 | 4 | 5 | |
|---|---|---|---|---|---|---|
| subitem not at all important | ○ | ○ | ○ | ○ | ◉ | essential |

### Does your paper address subitem 4b-i? *

Copy and paste relevant sections from the manuscript (include quotes in quotation marks "like this" to indicate direct quotes from your manuscript), or elaborate on this item by providing additional information not in the ms, or briefly explain why the item is not applicable/relevant for your study

Yes. "Upon receiving informed consent, REDCap generates a unique survey code linked to each participant's predefined email for the baseline and subsequent follow-up surveys. All data in REDCap are deidentified apart from participants' email address. Baseline data were collected using an iPad on the day of recruitment prior to randomization." The term "self-reported" was also used throughout the paper when reporting the

### 4b-ii) Report how institutional affiliations are displayed

Report how institutional affiliations are displayed to potential participants [on ehealth media], as affiliations with prestigious hospitals or universities may affect volunteer rates, use, and reactions with regards to an intervention.(Not a required item – describe only if this may bias results)

| | 1 | 2 | 3 | 4 | 5 | |
|---|---|---|---|---|---|---|
| subitem not at all important | ○ | ○ | ◉ | ○ | ○ | essential |

### Does your paper address subitem 4b-ii?

Copy and paste relevant sections from the manuscript (include quotes in quotation marks "like this" to indicate direct quotes from your manuscript), or elaborate on this item by providing additional information not in the ms, or briefly explain why the item is not applicable/relevant for your study

No. Institution affiliations did not affect recruitment. However, physician's advocacy may have influenced participants' decision to take part in the intervention, which was discussed in the discussion section. Yes

## 5) The interventions for each group with sufficient details to allow replication, including how and when they were actually administered

### 5-i) Mention names, credential, affiliations of the developers, sponsors, and owners

Mention names, credential, affiliations of the developers, sponsors, and owners [6] (if authors/evaluators are owners or developer of the software, this needs to be declared in a “Conflict of interest” section or mentioned elsewhere in the manuscript).

| | 1 | 2 | 3 | 4 | 5 | |
|---|---|---|---|---|---|---|
| subitem not at all important | ○ | ○ | ○ | ◉ | ○ | essential |

## Does your paper address subitem 5-i?

Copy and paste relevant sections from the manuscript (include quotes in quotation marks "like this" to indicate direct quotes from your manuscript), or elaborate on this item by providing additional information not in the ms, or briefly explain why the item is not applicable/relevant for your study

Yes. We mentioned that we used a third party commercial app and the rationale for choosing this app as our intervention. "Medisafe is a commercial, free medication management app available on both Android and iOS platforms. Its features include medication scheduling, reminder, tracking, data sharing, and medication adherence assessments. We selected a commercial app with evidence supporting its effectiveness to assess the feasibility of a smartphone app in promoting medication adherence in patients with type 2 diabetes." We had an agreement with Medisafe to use the app for research purposes but declare no financial

## 5-ii) Describe the history/development process

Describe the history/development process of the application and previous formative evaluations (e.g., focus groups, usability testing), as these will have an impact on adoption/use rates and help with interpreting results.

| | 1 | 2 | 3 | 4 | 5 | |
|---|---|---|---|---|---|---|
| subitem not at all important | ○ | ○ | ○ | ● | ○ | essential |

## Does your paper address subitem 5-ii?

Copy and paste relevant sections from the manuscript (include quotes in quotation marks "like this" to indicate direct quotes from your manuscript), or elaborate on this item by providing additional information not in the ms, or briefly explain why the item is not applicable/relevant for your study

Yes. We mentioned that we used a third party commercial app and the rationale for choosing this app as our intervention. "Medisafe is a commercial, free medication management app available on both Android and iOS platforms. Its features include medication scheduling, reminder, tracking, data sharing, and medication adherence assessments. We selected a commercial app with evidence supporting its effectiveness to assess the feasibility of a smartphone app in promoting medication adherence in patients with type 2 diabetes."

## 5-iii) Revisions and updating

Revisions and updating. Clearly mention the date and/or version number of the application/intervention (and comparator, if applicable) evaluated, or describe whether the intervention underwent major changes during the evaluation process, or whether the development and/or content was “frozen” during the trial. Describe dynamic components such as news feeds or changing content which may have an impact on the replicability of the intervention (for unexpected events see item 3b).

| | 1 | 2 | 3 | 4 | 5 | |
|---|---|---|---|---|---|---|
| subitem not at all important | ○ | ○ | ◉ | ○ | ○ | essential |

## Does your paper address subitem 5-iii?

Copy and paste relevant sections from the manuscript (include quotes in quotation marks "like this" to indicate direct quotes from your manuscript), or elaborate on this item by providing additional information not in the ms, or briefly explain why the item is not applicable/relevant for your study

No. The study did not undergo any major changes during the evaluation

## 5-iv) Quality assurance methods

Provide information on quality assurance methods to ensure accuracy and quality of information provided [1], if applicable.

| | 1 | 2 | 3 | 4 | 5 | |
|---|---|---|---|---|---|---|
| subitem not at all important | ○ | ○ | ◉ | ○ | ○ | essential |

## Does your paper address subitem 5-iv?

Copy and paste relevant sections from the manuscript (include quotes in quotation marks "like this" to indicate direct quotes from your manuscript), or elaborate on this item by providing additional information not in the ms, or briefly explain why the item is not applicable/relevant for your study

No. We mentioned in the limitations that "We were not able to observe app usage patterns of a few participants who changed smartphones during study follow-up. Medication adherence rates in the control group were also not tracked for comparison. Self-reported tools are subjective to a patient's own judgement and social desirability bias; hence, actual medication adherence may not be accurately reflected."

## 5-v) Ensure replicability by publishing the source code, and/or providing screenshots/screen-capture video, and/or providing flowcharts of the algorithms used

Ensure replicability by publishing the source code, and/or providing screenshots/screen-capture video, and/or providing flowcharts of the algorithms used. Replicability (i.e., other researchers should in principle be able to replicate the study) is a hallmark of scientific reporting.

| | 1 | 2 | 3 | 4 | 5 | |
|---|---|---|---|---|---|---|
| subitem not at all important | ○ | ○ | ○ | ◉ | ○ | essential |

## Does your paper address subitem 5-v?

Copy and paste relevant sections from the manuscript (include quotes in quotation marks "like this" to indicate direct quotes from your manuscript), or elaborate on this item by providing additional information not in the ms, or briefly explain why the item is not applicable/relevant for your study

This is not applicable as we did not develop the app. We provided a flowchart of the study procedures from recruitment to the end of data

## 5-vi) Digital preservation

Digital preservation: Provide the URL of the application, but as the intervention is likely to change or disappear over the course of the years; also make sure the intervention is archived (Internet Archive, webcitation.org, and/or publishing the source code or screenshots/videos alongside the article). As pages behind login screens cannot be archived, consider creating demo pages which are accessible without login.

| | 1 | 2 | 3 | 4 | 5 | |
|---|---|---|---|---|---|---|
| subitem not at all important | ○ | ○ | ● | ○ | ○ | essential |

## Does your paper address subitem 5-vi?

Copy and paste relevant sections from the manuscript (include quotes in quotation marks "like this" to indicate direct quotes from your manuscript), or elaborate on this item by providing additional information not in the ms, or briefly explain why the item is not applicable/relevant for your study

Yes. "https://www.medisafeapp.com/"

## 5-vii) Access

Access: Describe how participants accessed the application, in what setting/context, if they had to pay (or were paid) or not, whether they had to be a member of specific group. If known, describe how participants obtained “access to the platform and Internet” [1]. To ensure access for editors/reviewers/readers, consider to provide a “backdoor” login account or demo mode for reviewers/readers to explore the application (also important for archiving purposes, see vi).

| | 1 | 2 | 3 | 4 | 5 | |
|---|---|---|---|---|---|---|
| subitem not at all important | ○ | ○ | ○ | ● | ○ | essential |

## Does your paper address subitem 5-vii? *

Copy and paste relevant sections from the manuscript (include quotes in quotation marks "like this" to indicate direct quotes from your manuscript), or elaborate on this item by providing additional information not in the ms, or briefly explain why the item is not applicable/relevant for your study

Yes. "Participants were asked to use the app freely outside the health care setting and add the research group as a “Medfriend” for their medication-taking patterns to be observed. Medisafe is a commercial, free medication management app available on both Android and iOS platforms."

## 5-viii) Mode of delivery, features/functionalities/components of the intervention and comparator, and the theoretical framework

Describe mode of delivery, features/functionalities/components of the intervention and comparator, and the theoretical framework [6] used to design them (instructional strategy [1], behaviour change techniques, persuasive features, etc., see e.g., [7, 8] for terminology). This includes an in-depth description of the content (including where it is coming from and who developed it) [1],” whether [and how] it is tailored to individual circumstances and allows users to track their progress and receive feedback” [6]. This also includes a description of communication delivery channels and – if computer-mediated communication is a component – whether communication was synchronous or asynchronous [6]. It also includes information on presentation strategies [1], including page design principles, average amount of text on pages, presence of hyperlinks to other resources, etc. [1].

| | 1 | 2 | 3 | 4 | 5 | |
|---|---|---|---|---|---|---|
| subitem not at all important | ○ | ○ | ◉ | ○ | ○ | essential |

## Does your paper address subitem 5-viii? *

Copy and paste relevant sections from the manuscript (include quotes in quotation marks "like this" to indicate direct quotes from your manuscript), or elaborate on this item by providing additional information not in the ms, or briefly explain why the item is not applicable/relevant for your study

Yes. "Medisafe is a commercial, free medication management app available on both Android and iOS platforms. Its features include medication scheduling, reminder, tracking, data sharing, and medication adherence assessments. We selected a commercial app with evidence supporting its effectiveness to assess the feasibility of a smartphone app in promoting medication adherence in patients with type 2 diabetes. "

## 5-ix) Describe use parameters

Describe use parameters (e.g., intended "doses" and optimal timing for use). Clarify what instructions or recommendations were given to the user, e.g., regarding timing, frequency, heaviness of use, if any, or was the intervention used ad libitum.

| | 1 | 2 | 3 | 4 | 5 | |
|---|---|---|---|---|---|---|
| subitem not at all important | ○ | ○ | ○ | ○ | ◉ | essential |

## Does your paper address subitem 5-ix?

Copy and paste relevant sections from the manuscript (include quotes in quotation marks "like this" to indicate direct quotes from your manuscript), or elaborate on this item by providing additional information not in the ms, or briefly explain why the item is not applicable/relevant for your study

Yes. "Participants were asked to use the app freely outside the health care setting and add the research group as a "Medfriend" for their medication-taking patterns to be observed." "The research team, as a "Medfriend," did not interact with the participant during the follow-up period."

## 5-x) Clarify the level of human involvement

Clarify the level of human involvement (care providers or health professionals, also technical assistance) in the e-intervention or as co-intervention (detail number and expertise of professionals involved, if any, as well as "type of assistance offered, the timing and frequency of the support, how it is initiated, and the medium by which the assistance is delivered". It may be necessary to distinguish between the level of human involvement required for the trial, and the level of human involvement required for a routine application outside of a RCT setting (discuss under item 21 – generalizability).

| | 1 | 2 | 3 | 4 | 5 | |
|---|---|---|---|---|---|---|
| subitem not at all important | ○ | ○ | ○ | ◉ | ○ | essential |

## Does your paper address subitem 5-x?

Copy and paste relevant sections from the manuscript (include quotes in quotation marks "like this" to indicate direct quotes from your manuscript), or elaborate on this item by providing additional information not in the ms, or briefly explain why the item is not applicable/relevant for your study

Yes. We mentioned that the participants were referred from four endocrinologists and that "participants were assisted by the researchers to download the Medisafe app on their personal smartphone, set their medication schedule and reminder on the app, and to use the app." We also mentioned that "The research team, as a "Medfriend," did not interact with the participant during the follow-up period." As this is a feasibility trial, the number of endocrinologists and researchers involved were not indicative of actual human involvement required. We provided recommendations for a study scale up to a full trial.

## 5-xi) Report any prompts/reminders used

Report any prompts/reminders used: Clarify if there were prompts (letters, emails, phone calls, SMS) to use the application, what triggered them, frequency etc. It may be necessary to distinguish between the level of prompts/reminders required for the trial, and the level of prompts/reminders for a routine application outside of a RCT setting (discuss under item 21 – generalizability).

| | 1 | 2 | 3 | 4 | 5 | |
|---|---|---|---|---|---|---|
| subitem not at all important | ○ | ○ | ○ | ○ | ◉ | essential |

## Does your paper address subitem 5-xi? *

Copy and paste relevant sections from the manuscript (include quotes in quotation marks "like this" to indicate direct quotes from your manuscript), or elaborate on this item by providing additional information not in the ms, or briefly explain why the item is not applicable/relevant for your study

Yes. "The intervention group participants were reminded via email to complete two intermediate and one final online survey at 4-week intervals during the 12-week follow-up period. Control group participants were instructed to complete only one online survey at the end of the 12-week follow-up period. All follow-up surveys were conducted online via a unique link sent to the participants' email address. Each unique survey link was accessible for a maximum of 14 days or until the participant completed the survey. Participants in both groups were reminded by calling them on their mobile phone to complete the final survey if no

## 5-xii) Describe any co-interventions (incl. training/support)

Describe any co-interventions (incl. training/support): Clearly state any interventions that are provided in addition to the targeted eHealth intervention, as ehealth intervention may not be designed as stand-alone intervention. This includes training sessions and support [1]. It may be necessary to distinguish between the level of training required for the trial, and the level of training for a routine application outside of a RCT setting (discuss under item 21 – generalizability.

| | 1 | 2 | 3 | 4 | 5 | |
|---|---|---|---|---|---|---|
| subitem not at all important | ○ | ○ | ○ | ● | ○ | essential |

## Does your paper address subitem 5-xii? *

Copy and paste relevant sections from the manuscript (include quotes in quotation marks "like this" to indicate direct quotes from your manuscript), or elaborate on this item by providing additional information not in the ms, or briefly explain why the item is not applicable/relevant for your study

No. Other than helping the participants to download the app and set up a medication reminder schedule, there were no other training sessions.

## 6a) Completely defined pre-specified primary and secondary outcome measures, including how and when they were assessed

### Does your paper address CONSORT subitem 6a? *

Copy and paste relevant sections from the manuscript (include quotes in quotation marks "like this" to indicate direct quotes from your manuscript), or elaborate on this item by providing additional information not in the ms, or briefly explain why the item is not applicable/relevant for your study

Yes. The primary and secondary outcomes, and their data collection methods were specified in the manuscript.

### 6a-i) Online questionnaires: describe if they were validated for online use and apply CHERRIES items to describe how the questionnaires were designed/deployed

If outcomes were obtained through online questionnaires, describe if they were validated for online use and apply CHERRIES items to describe how the questionnaires were designed/deployed [9].

| | 1 | 2 | 3 | 4 | 5 | |
|---|---|---|---|---|---|---|
| subitem not at all important | ○ | ○ | ○ | ◉ | ○ | essential |

### Does your paper address subitem 6a-i?

Copy and paste relevant sections from manuscript text

We have cross checked the manuscript with the CHERRIES items. Sections that were applicable were reported in the manuscript.

## 6a-ii) Describe whether and how “use” (including intensity of use/dosage) was defined/measured/monitored

Describe whether and how “use” (including intensity of use/dosage) was defined/measured/monitored (logins, logfile analysis, etc.). Use/adoption metrics are important process outcomes that should be reported in any ehealth trial.

| | 1 | 2 | 3 | 4 | 5 | |
|---|---|---|---|---|---|---|
| subitem not at all important | ○ | ○ | ○ | ◉ | ○ | essential |

## Does your paper address subitem 6a-ii?

Copy and paste relevant sections from manuscript text

Yes. "Another measure of feasibility is adherence to trial participation, which was assessed by observing intervention group participants’ interaction with the app throughout the intervention through the “Medfriend” feature of the app."

## 6a-iii) Describe whether, how, and when qualitative feedback from participants was obtained

Describe whether, how, and when qualitative feedback from participants was obtained (e.g., through emails, feedback forms, interviews, focus groups).

| | 1 | 2 | 3 | 4 | 5 | |
|---|---|---|---|---|---|---|
| subitem not at all important | ○ | ○ | ○ | ◉ | ○ | essential |

## Does your paper address subitem 6a-iii?

Copy and paste relevant sections from manuscript text

Yes. "We collected come participant feedback with the online satisfaction survey and while handing out vouchers to participants who completed the online survey(s)."

## 6b) Any changes to trial outcomes after the trial commenced, with reasons

### Does your paper address CONSORT subitem 6b? *

Copy and paste relevant sections from the manuscript (include quotes in quotation marks "like this" to indicate direct quotes from your manuscript), or elaborate on this item by providing additional information not in the ms, or briefly explain why the item is not applicable/relevant for your study

We asked participants to indicate their level of medication adherence on a visual analogue scale pre- and post- study. However, this outcome was not reported in the manuscript as we found this measure to be inaccurate during recruitment. For example, many participants indicated that they sometimes forget to take their medication in the ASK-12 questionnaire, but would still indicate that they took 100% of their medication in the VAS. When prompted further, we realized that many older participants had difficulty indicating the percentage of medication they took. We initially collected data on the VAS due to its ease of administration and potential to cross reference with the ASK-12 scale.

## 7a) How sample size was determined

NPT: When applicable, details of whether and how the clustering by care provides or centers was addressed

### 7a-i) Describe whether and how expected attrition was taken into account when calculating the sample size

Describe whether and how expected attrition was taken into account when calculating the sample size.

| | 1 | 2 | 3 | 4 | 5 | |
|---|---|---|---|---|---|---|
| subitem not at all important | ○ | ○ | ○ | ◉ | ○ | essential |

## Does your paper address subitem 7a-i?

Copy and paste relevant sections from manuscript title (include quotes in quotation marks "like this" to indicate direct quotes from your manuscript), or elaborate on this item by providing additional information not in the ms, or briefly explain why the item is not applicable/relevant for your study

We estimated the sample size for a trial as we could not anticipate the recruitment rate. This was one of the reason we conducted a feasibility pilot study.

## 7b) When applicable, explanation of any interim analyses and stopping guidelines

## Does your paper address CONSORT subitem 7b? *

Copy and paste relevant sections from the manuscript (include quotes in quotation marks "like this" to indicate direct quotes from your manuscript), or elaborate on this item by providing additional information not in the ms, or briefly explain why the item is not applicable/relevant for your study

There was no interim analyses and stopping guidelines.

## 8a) Method used to generate the random allocation sequence

NPT: When applicable, how care providers were allocated to each trial group

## Does your paper address CONSORT subitem 8a? *

Copy and paste relevant sections from the manuscript (include quotes in quotation marks "like this" to indicate direct quotes from your manuscript), or elaborate on this item by providing additional information not in the ms, or briefly explain why the item is not applicable/relevant for your study

Yes. "Block randomization (blocks of four) was conducted to ensure a balanced allocation, since we could not anticipate the final sample size. Eligible participants were asked to draw a card from a box with two “intervention” and two “control” cards, which were reset after all four cards were drawn."

## 8b) Type of randomisation; details of any restriction (such as blocking and block size)

### Does your paper address CONSORT subitem 8b? *

Copy and paste relevant sections from the manuscript (include quotes in quotation marks "like this" to indicate direct quotes from your manuscript), or elaborate on this item by providing additional information not in the ms, or briefly explain why the item is not applicable/relevant for your study

Yes. "Block randomization (blocks of four) was conducted to ensure a balanced allocation."

## 9) Mechanism used to implement the random allocation sequence (such as sequentially numbered containers), describing any steps taken to conceal the sequence until interventions were assigned

### Does your paper address CONSORT subitem 9? *

Copy and paste relevant sections from the manuscript (include quotes in quotation marks "like this" to indicate direct quotes from your manuscript), or elaborate on this item by providing additional information not in the ms, or briefly explain why the item is not applicable/relevant for your study

Yes. "Eligible participants were asked to draw a card from a box with two “intervention” and two “control” cards, which were reset after all four cards were drawn."

## 10) Who generated the random allocation sequence, who enrolled participants, and who assigned participants to interventions

## Does your paper address CONSORT subitem 10? *

Copy and paste relevant sections from the manuscript (include quotes in quotation marks "like this" to indicate direct quotes from your manuscript), or elaborate on this item by providing additional information not in the ms, or briefly explain why the item is not applicable/relevant for your study

Yes. The researchers recruited the participants and block randomized them. This was described in the methods section.

## 11a) If done, who was blinded after assignment to interventions (for example, participants, care providers, those assessing outcomes) and how

NPT: Whether or not administering co-interventions were blinded to group assignment

## 11a-i) Specify who was blinded, and who wasn't

Specify who was blinded, and who wasn't. Usually, in web-based trials it is not possible to blind the participants [1, 3] (this should be clearly acknowledged), but it may be possible to blind outcome assessors, those doing data analysis or those administering co-interventions (if any).

| | 1 | 2 | 3 | 4 | 5 | |
|---|---|---|---|---|---|---|
| subitem not at all important | ○ | ○ | ○ | ○ | ● | essential |

## Does your paper address subitem 11a-i? *

Copy and paste relevant sections from the manuscript (include quotes in quotation marks "like this" to indicate direct quotes from your manuscript), or elaborate on this item by providing additional information not in the ms, or briefly explain why the item is not applicable/relevant for your study

Yes. "The clinical care team was blinded from the study. Participants were only partially blinded, as we had to explain the purpose of the study before randomization. The name of the app was not revealed to participants unless they were randomized into the intervention group or screened out of the study."

## 11a-ii) Discuss e.g., whether participants knew which intervention was the “intervention of interest” and which one was the “comparator”

Informed consent procedures (4a-ii) can create biases and certain expectations - discuss e.g., whether participants knew which intervention was the “intervention of interest” and which one was the “comparator”.

| | 1 | 2 | 3 | 4 | 5 | |
|---|---|---|---|---|---|---|
| subitem not at all important | ○ | ○ | ○ | ◉ | ○ | essential |

## Does your paper address subitem 11a-ii?

Copy and paste relevant sections from the manuscript (include quotes in quotation marks "like this" to indicate direct quotes from your manuscript), or elaborate on this item by providing additional information not in the ms, or briefly explain why the item is not applicable/relevant for your study

Yes. Participants knew which group they were allocated to as we had to explain that they may or may not need to use an app for the study. "Participants were only partially blinded, as we had to explain the purpose of the study before randomization. The name of the app was not revealed to participants unless they were randomized into the intervention group or screened out of the study."

## 11b) If relevant, description of the similarity of interventions

(this item is usually not relevant for ehealth trials as it refers to similarity of a placebo or sham intervention to a active medication/intervention)

## Does your paper address CONSORT subitem 11b? *

Copy and paste relevant sections from the manuscript (include quotes in quotation marks "like this" to indicate direct quotes from your manuscript), or elaborate on this item by providing additional information not in the ms, or briefly explain why the item is not applicable/relevant for your study

N.A.

## 12a) Statistical methods used to compare groups for primary and secondary outcomes

NPT: When applicable, details of whether and how the clustering by care providers or centers was addressed

### Does your paper address CONSORT subitem 12a? *

Copy and paste relevant sections from the manuscript (include quotes in quotation marks "like this" to indicate direct quotes from your manuscript), or elaborate on this item by providing additional information not in the ms, or briefly explain why the item is not applicable/relevant for your study

Yes. "Descriptive analyses were used for baseline comparisons, and linear regressions, controlled for baseline imbalances, were used to compare the pretest and posttest change scores. All statistical assumptions were checked to ensure the accuracy of analyses. Statistical significance was set at P<.05. SPSS [computer software] (Version 22. Armonk, NY: IBM Corp) was used for all statistical analyses."

### 12a-i) Imputation techniques to deal with attrition / missing values

Imputation techniques to deal with attrition / missing values: Not all participants will use the intervention/comparator as intended and attrition is typically high in ehealth trials. Specify how participants who did not use the application or dropped out from the trial were treated in the statistical analysis (a complete case analysis is strongly discouraged, and simple imputation techniques such as LOCF may also be problematic [4]).

| | 1 | 2 | 3 | 4 | 5 | |
|---|---|---|---|---|---|---|
| subitem not at all important | ○ | ○ | ○ | ◉ | ○ | essential |

## Does your paper address subitem 12a-i? *

Copy and paste relevant sections from the manuscript (include quotes in quotation marks "like this" to indicate direct quotes from your manuscript), or elaborate on this item by providing additional information not in the ms, or briefly explain why the item is not applicable/relevant for your study

Yes. We did not perform any imputation as the attrition rate was not high. "The intention-to-treat approach was used to analyze the data. We excluded participants who did not complete the final survey due to the lack of poststudy data for pre-post comparison. Intervention group participants who stopped using the app during the study and control group participants who used an app to manage their medications during the study follow-up period were included in the analysis."

## 12b) Methods for additional analyses, such as subgroup analyses and adjusted analyses

## Does your paper address CONSORT subitem 12b? *

Copy and paste relevant sections from the manuscript (include quotes in quotation marks "like this" to indicate direct quotes from your manuscript), or elaborate on this item by providing additional information not in the ms, or briefly explain why the item is not applicable/relevant for your study

Yes. "Descriptive analyses were used for baseline comparisons, and linear regressions, controlled for baseline imbalances, were used to compare the pretest and posttest change scores. All statistical assumptions were checked to ensure the accuracy of analyses."

## X26) REB/IRB Approval and Ethical Considerations [recommended as subheading under "Methods"] (not a CONSORT item)

## X26-i) Comment on ethics committee approval

| | 1 | 2 | 3 | 4 | 5 | |
|---|---|---|---|---|---|---|
| subitem not at all important | ○ | ○ | ○ | ○ | ◉ | essential |

## Does your paper address subitem X26-i?

Copy and paste relevant sections from the manuscript (include quotes in quotation marks "like this" to indicate direct quotes from your manuscript), or elaborate on this item by providing additional information not in the ms, or briefly explain why the item is not applicable/relevant for your study

Yes. "This study was approved by the SingHealth Centralised Institutional Review Board (Reference: 2018/2563) and the Nanyang Technological University Institutional Review Board (Reference: IRB-2018-09-029) in Singapore."

## x26-ii) Outline informed consent procedures

Outline informed consent procedures e.g., if consent was obtained offline or online (how? Checkbox, etc.?), and what information was provided (see 4a-ii). See [6] for some items to be included in informed consent documents.

| | 1 | 2 | 3 | 4 | 5 | |
|---|---|---|---|---|---|---|
| subitem not at all important | ○ | ○ | ◉ | ○ | ○ | essential |

## Does your paper address subitem X26-ii?

Copy and paste relevant sections from the manuscript (include quotes in quotation marks "like this" to indicate direct quotes from your manuscript), or elaborate on this item by providing additional information not in the ms, or briefly explain why the item is not applicable/relevant for your study

Yes."The patient was termed a research participant once the informed consent document was signed. At the point of consent seeking, researchers explained to potential participants that they may or may not be selected for the study, depending on their eligibility, which can only be determined after they respond to the baseline questionnaire. Informed consent was collected with a printed hardcopy forms."

## X26-iii) Safety and security procedures

Safety and security procedures, incl. privacy considerations, and any steps taken to reduce the likelihood or detection of harm (e.g., education and training, availability of a hotline)

| | 1 | 2 | 3 | 4 | 5 | |
|---|---|---|---|---|---|---|
| subitem not at all important | ○ | ○ | ◉ | ○ | ○ | essential |

## Does your paper address subitem X26-iii?

Copy and paste relevant sections from the manuscript (include quotes in quotation marks "like this" to indicate direct quotes from your manuscript), or elaborate on this item by providing additional information not in the ms, or briefly explain why the item is not applicable/relevant for your study

Yes. "REDCap is a secure, Web-based software platform designed to support data capture for research studies, providing an intuitive interface for validated data capture; audit trails for tracking data manipulation and export procedures; automated export procedures for seamless data downloads to common statistical packages; and procedures for data integration and interoperability with external sources. Upon receiving informed consent, REDCap generates a unique survey code linked to each participant's predefined email for the baseline and subsequent follow-up surveys. All data in REDCap are deidentified apart from participants' email address. Baseline data were collected using an iPad on the day of recruitment prior to randomization."

# RESULTS

## 13a) For each group, the numbers of participants who were randomly assigned, received intended treatment, and were analysed for the primary outcome

NPT: The number of care providers or centers performing the intervention in each group and the number of patients treated by each care provider in each center

## Does your paper address CONSORT subitem 13a? *

Copy and paste relevant sections from the manuscript (include quotes in quotation marks "like this" to indicate direct quotes from your manuscript), or elaborate on this item by providing additional information not in the ms, or briefly explain why the item is not applicable/relevant for your study

Yes. These information were illustrated in Figure 2 and described in the Results - Recruitment section.

## 13b) For each group, losses and exclusions after randomisation, together with reasons

## Does your paper address CONSORT subitem 13b? (NOTE: Preferably, this is shown in a CONSORT flow diagram) *

Copy and paste relevant sections from the manuscript (include quotes in quotation marks "like this" to indicate direct quotes from your manuscript), or elaborate on this item by providing additional information not in the ms, or briefly explain why the item is not applicable/relevant for your study

Yes. Figure 2 details the participant flow from recruitment to data analyses. The reasons for study decline, exclusions and attrition were detailed in the diagram.

## 13b-i) Attrition diagram

Strongly recommended: An attrition diagram (e.g., proportion of participants still logging in or using the intervention/comparator in each group plotted over time, similar to a survival curve) or other figures or tables demonstrating usage/dose/engagement.

| | 1 | 2 | 3 | 4 | 5 | |
|---|---|---|---|---|---|---|
| subitem not at all important | ○ | ○ | ● | ○ | ○ | essential |

## Does your paper address subitem 13b-i?

Copy and paste relevant sections from the manuscript or cite the figure number if applicable (include quotes in quotation marks "like this" to indicate direct quotes from your manuscript), or elaborate on this item by providing additional information not in the ms, or briefly explain why the item is not applicable/relevant for your study

Yes. Figure 2 details the participant flow from recruitment to data analyses. The reasons for attrition in both groups were detailed in the diagram.

## 14a) Dates defining the periods of recruitment and follow-up

## Does your paper address CONSORT subitem 14a? *

Copy and paste relevant sections from the manuscript (include quotes in quotation marks "like this" to indicate direct quotes from your manuscript), or elaborate on this item by providing additional information not in the ms, or briefly explain why the item is not applicable/relevant for your study

Yes. "Participants were recruited over 10 weeks from September to November 2018 at a tertiary diabetes specialist outpatient center". We also specified in the manuscript that the participants were each followed-up for 12-weeks outside the healthcare setting. The schedule of outcome measurements was shown in figure 1.

## 14a-i) Indicate if critical "secular events" fell into the study period

Indicate if critical "secular events" fell into the study period, e.g., significant changes in Internet resources available or "changes in computer hardware or Internet delivery resources"

| | 1 | 2 | 3 | 4 | 5 | |
|---|---|---|---|---|---|---|
| subitem not at all important | ○ | ○ | ○ | ● | ○ | essential |

## Does your paper address subitem 14a-i?

Copy and paste relevant sections from the manuscript (include quotes in quotation marks "like this" to indicate direct quotes from your manuscript), or elaborate on this item by providing additional information not in the ms, or briefly explain why the item is not applicable/relevant for your study

There were no critical eHealth events that occured during the study period. However, there were a few national festive periods (ie, Diwali, Chirstmas, Chinese New Year) which may have affected the eating patterns of the participants. Hence, the health outomes of interest may have been affected. This was mentioned in the discussion section: "We observed increased HbA1c levels in both groups, which is attributed to the follow-up period falling within a few holiday seasons (ie, Diwali, Christmas, and Chinese New Year) where festive feasting in Asian

## 14b) Why the trial ended or was stopped (early)

## Does your paper address CONSORT subitem 14b? *

Copy and paste relevant sections from the manuscript (include quotes in quotation marks "like this" to indicate direct quotes from your manuscript), or elaborate on this item by providing additional information not in the ms, or briefly explain why the item is not applicable/relevant for your study

N.A. The trial did not stop or end early.

## 15) A table showing baseline demographic and clinical characteristics for each group

NPT: When applicable, a description of care providers (case volume, qualification, expertise, etc.) and centers (volume) in each group

## Does your paper address CONSORT subitem 15? *

Copy and paste relevant sections from the manuscript (include quotes in quotation marks "like this" to indicate direct quotes from your manuscript), or elaborate on this item by providing additional information not in the ms, or briefly explain why the item is not applicable/relevant for your study

Yes, the baseline profile of participants were collected the reported.

## 15-i) Report demographics associated with digital divide issues

In ehealth trials it is particularly important to report demographics associated with digital divide issues, such as age, education, gender, social-economic status, computer/Internet/ehealth literacy of the participants, if known.

| | 1 | 2 | 3 | 4 | 5 | |
|---|---|---|---|---|---|---|
| subitem not at all important | ○ | ○ | ○ | ○ | ◉ | essential |

## Does your paper address subitem 15-i? *

Copy and paste relevant sections from the manuscript (include quotes in quotation marks "like this" to indicate direct quotes from your manuscript), or elaborate on this item by providing additional information not in the ms, or briefly explain why the item is not applicable/relevant for your study

Yes. "The baseline characteristics of patients included in the analysis are shown in Table 1."

## 16) For each group, number of participants (denominator) included in each analysis and whether the analysis was by original assigned groups

## 16-i) Report multiple “denominators” and provide definitions

Report multiple “denominators” and provide definitions: Report N’s (and effect sizes) “across a range of study participation [and use] thresholds” [1], e.g., N exposed, N consented, N used more than x times, N used more than y weeks, N participants “used” the intervention/comparator at specific pre-defined time points of interest (in absolute and relative numbers per group). Always clearly define “use” of the intervention.

| | 1 | 2 | 3 | 4 | 5 | |
|---|---|---|---|---|---|---|
| subitem not at all important | ○ | ○ | ○ | ● | ○ | essential |

## Does your paper address subitem 16-i? *

Copy and paste relevant sections from the manuscript (include quotes in quotation marks "like this" to indicate direct quotes from your manuscript), or elaborate on this item by providing additional information not in the ms, or briefly explain why the item is not applicable/relevant for your study

Yes. Multiple denominators were reported for different analyses. Figure 2 shows the participant flow from recruitment to the end of data collection.

## 16-ii) Primary analysis should be intent-to-treat

Primary analysis should be intent-to-treat, secondary analyses could include comparing only “users”, with the appropriate caveats that this is no longer a randomized sample (see 18-i).

| | 1 | 2 | 3 | 4 | 5 | |
|---|---|---|---|---|---|---|
| subitem not at all important | ○ | ○ | ○ | ● | ○ | essential |

## Does your paper address subitem 16-ii?

Copy and paste relevant sections from the manuscript (include quotes in quotation marks "like this" to indicate direct quotes from your manuscript), or elaborate on this item by providing additional information not in the ms, or briefly explain why the item is not applicable/relevant for your study

Yes. "The intention-to-treat approach was used to analyze the data. We excluded participants who did not complete the final survey due to the lack of poststudy data for pre-post comparison. Intervention group participants who stopped using the app during the study and control group participants who used an app to manage their medications during the study follow-up period were included in the analysis."

## 17a) For each primary and secondary outcome, results for each group, and the estimated effect size and its precision (such as 95% confidence interval)

## Does your paper address CONSORT subitem 17a? *

Copy and paste relevant sections from the manuscript (include quotes in quotation marks "like this" to indicate direct quotes from your manuscript), or elaborate on this item by providing additional information not in the ms, or briefly explain why the item is not applicable/relevant for your study

Yes. The estimated effect size and its precision for each group were reported in Table 2.

## 17a-i) Presentation of process outcomes such as metrics of use and intensity of use

In addition to primary/secondary (clinical) outcomes, the presentation of process outcomes such as metrics of use and intensity of use (dose, exposure) and their operational definitions is critical. This does not only refer to metrics of attrition (13-b) (often a binary variable), but also to more continuous exposure metrics such as "average session length". These must be accompanied by a technical description how a metric like a "session" is defined (e.g., timeout after idle time) [1] (report under item 6a).

| | 1 | 2 | 3 | 4 | 5 | |
|---|---|---|---|---|---|---|
| subitem not at all important | ○ | ○ | ● | ○ | ○ | essential |

## Does your paper address subitem 17a-i?

Copy and paste relevant sections from the manuscript (include quotes in quotation marks "like this" to indicate direct quotes from your manuscript), or elaborate on this item by providing additional information not in the ms, or briefly explain why the item is not applicable/relevant for your study

Yes. "Another measure of feasibility is adherence to trial participation, which was assessed by observing intervention group participants' interaction with the app throughout the intervention through the "Medfriend" feature of the app."

## 17b) For binary outcomes, presentation of both absolute and relative effect sizes is recommended

## Does your paper address CONSORT subitem 17b? *

Copy and paste relevant sections from the manuscript (include quotes in quotation marks "like this" to indicate direct quotes from your manuscript), or elaborate on this item by providing additional information not in the ms, or briefly explain why the item is not applicable/relevant for your study

The main outcomes were not binary. Hence, the absolute effect size was not reported.

## 18) Results of any other analyses performed, including subgroup analyses and adjusted analyses, distinguishing pre-specified from exploratory

## Does your paper address CONSORT subitem 18? *

Copy and paste relevant sections from the manuscript (include quotes in quotation marks "like this" to indicate direct quotes from your manuscript), or elaborate on this item by providing additional information not in the ms, or briefly explain why the item is not applicable/relevant for your study

Yes. Adjusted outcomes were presented. Subgroup analyses was not presented as the sample size of the subgroups were too smal to make any meaningful conclusions.

## 18-i) Subgroup analysis of comparing only users

A subgroup analysis of comparing only users is not uncommon in ehealth trials, but if done, it must be stressed that this is a self-selected sample and no longer an unbiased sample from a randomized trial (see 16-iii).

| | 1 | 2 | 3 | 4 | 5 | |
|---|---|---|---|---|---|---|
| subitem not at all important | ○ | ○ | ◉ | ○ | ○ | essential |

## Does your paper address subitem 18-i?

Copy and paste relevant sections from the manuscript (include quotes in quotation marks "like this" to indicate direct quotes from your manuscript), or elaborate on this item by providing additional information not in the ms, or briefly explain why the item is not applicable/relevant for your study

Yes. Attitude, Perception, Satisfaction and app usage patterns were assessed only in the intervention group as these are intervention related

## 19) All important harms or unintended effects in each group

(for specific guidance see CONSORT for harms)

## Does your paper address CONSORT subitem 19? *

Copy and paste relevant sections from the manuscript (include quotes in quotation marks "like this" to indicate direct quotes from your manuscript), or elaborate on this item by providing additional information not in the ms, or briefly explain why the item is not applicable/relevant for your study

Yes. The Medisafe app caused annoyance to some participants. "use of the app did not reduce medication management stress in 34% of the respondents, and 80% of the respondents found the reminder notification

## 19-i) Include privacy breaches, technical problems

Include privacy breaches, technical problems. This does not only include physical “harm” to participants, but also incidents such as perceived or real privacy breaches [1], technical problems, and other unexpected/unintended incidents. “Unintended effects” also includes unintended positive effects [2].

| | 1 | 2 | 3 | 4 | 5 | |
|---|---|---|---|---|---|---|
| subitem not at all important | ○ | ○ | ○ | ◉ | ○ | essential |

## Does your paper address subitem 19-i?

Copy and paste relevant sections from the manuscript (include quotes in quotation marks "like this" to indicate direct quotes from your manuscript), or elaborate on this item by providing additional information not in the ms, or briefly explain why the item is not applicable/relevant for your study

Yes. The Medisafe app caused annoyance to some participants. "use of the app did not reduce medication management stress in 34% of the respondents, and 80% of the respondents found the reminder notification annoying." There were no privacy breaches. The technical problems participants faced were mostly due to low digital literacy. These issues were mentioned in the discussion section of the manuscript.

## 19-ii) Include qualitative feedback from participants or observations from staff/researchers

Include qualitative feedback from participants or observations from staff/researchers, if available, on strengths and shortcomings of the application, especially if they point to unintended/unexpected effects or uses. This includes (if available) reasons for why people did or did not use the application as intended by the developers.

| | 1 | 2 | 3 | 4 | 5 | |
|---|---|---|---|---|---|---|
| subitem not at all important | ○ | ○ | ○ | ◉ | ○ | essential |

## Does your paper address subitem 19-ii?

Copy and paste relevant sections from the manuscript (include quotes in quotation marks "like this" to indicate direct quotes from your manuscript), or elaborate on this item by providing additional information not in the ms, or briefly explain why the item is not applicable/relevant for your study

Yes. Participants' feedback was mentioned in the Results - Acceptability of the Medication Management Smartphone App section. "Two participants would like to add their spouses as a “Medfriend” but could not do so, as the free version only allowed the addition of one “Medfriend” (ie, the study team). Other feedback include suggestions to incorporate the doctor’s appointment scheduling and other diabetes self-management features, simplifying the app interface, educating participants on manipulating the settings, and integrating some of the hospital’s services with the app. Although Medisafe is a third-party app, patients would prefer integrating all health services into a one-stop reliable and personalized platform."

# DISCUSSION

## 22) Interpretation consistent with results, balancing benefits and harms, and considering other relevant evidence

NPT: In addition, take into account the choice of the comparator, lack of or partial blinding, and unequal expertise of care providers or centers in each group

## 22-i) Restate study questions and summarize the answers suggested by the data, starting with primary outcomes and process outcomes (use)

Restate study questions and summarize the answers suggested by the data, starting with primary outcomes and process outcomes (use).

| | 1 | 2 | 3 | 4 | 5 | |
|---|---|---|---|---|---|---|
| subitem not at all important | ○ | ◉ | ○ | ○ | ○ | essential |

## Does your paper address subitem 22-i? *

Copy and paste relevant sections from the manuscript (include quotes in quotation marks "like this" to indicate direct quotes from your manuscript), or elaborate on this item by providing additional information not in the ms, or briefly explain why the item is not applicable/relevant for your study

Yes. "We established the feasibility of using a smartphone app to improve medication adherence in patients with type 2 diabetes managed at a public diabetes specialist outpatient center in Singapore through a pilot study." "We observed significantly lower self-reported barriers to medication adherence in the intervention group compared with the control group but no improvement in the HbA1c level."

## 22-ii) Highlight unanswered new questions, suggest future research

Highlight unanswered new questions, suggest future research.

| | 1 | 2 | 3 | 4 | 5 | |
|---|---|---|---|---|---|---|
| subitem not at all important | ○ | ○ | ◉ | ○ | ○ | essential |

## Does your paper address subitem 22-ii?

Copy and paste relevant sections from the manuscript (include quotes in quotation marks "like this" to indicate direct quotes from your manuscript), or elaborate on this item by providing additional information not in the ms, or briefly explain why the item is not applicable/relevant for your study

Yes. "Our findings suggest that should a full randomized controlled trial be conducted, a five-fold scale-up is required to achieve full trial power under the same conditions. This can be achieved with the involvement of more physicians, more study sites, or a longer recruitment period. Future studies should assess factors that could enhance the usability of apps in older adults who are less technologically savvy. The app usage behavior of different patient subgroups and interaction between various diabetes app features can also be explored. "

## 20) Trial limitations, addressing sources of potential bias, imprecision, and, if relevant, multiplicity of analyses

### 20-i) Typical limitations in ehealth trials

Typical limitations in ehealth trials: Participants in ehealth trials are rarely blinded. Ehealth trials often look at a multiplicity of outcomes, increasing risk for a Type I error. Discuss biases due to non-use of the intervention/usability issues, biases through informed consent procedures, unexpected events.

| | 1 | 2 | 3 | 4 | 5 | |
|---|---|---|---|---|---|---|
| subitem not at all important | ○ | ○ | ○ | ○ | ◉ | essential |

### Does your paper address subitem 20-i? *

Copy and paste relevant sections from the manuscript (include quotes in quotation marks "like this" to indicate direct quotes from your manuscript), or elaborate on this item by providing additional information not in the ms, or briefly explain why the item is not applicable/relevant for your study

Yes. "There were limitations to the study. We were not able to observe app usage patterns of a few participants who changed smartphones during study follow-up. Medication adherence rates in the control group were also not tracked for comparison. Self-reported tools are subjective to a patient's own judgement and social desirability bias; hence, actual medication adherence may not be accurately reflected. We observed patients who over- and underreported their medication adherence status and problems with survey interpretation. For example, when the researchers verbally asked (at baseline), "How likely do you think your diabetes will worsen in the next few years?" a few participants answered "I hope it will not worsen" instead of choosing a Likert scale response. The study may not be generalizable to all people with diabetes, as tertiary specialist outpatient clinics are likely to manage more complex cases that cannot be managed in the primary care setting. Lastly, contamination may have occurred when the control group participants were exposed to the idea of using an app for type 2 diabetes medication management."

## 21) Generalisability (external validity, applicability) of the trial findings

NPT: External validity of the trial findings according to the intervention, comparators, patients, and care providers or centers involved in the trial

### 21-i) Generalizability to other populations

Generalizability to other populations: In particular, discuss generalizability to a general Internet population, outside of a RCT setting, and general patient population, including applicability of the study results for other organizations

| | 1 | 2 | 3 | 4 | 5 | |
|---|---|---|---|---|---|---|
| subitem not at all important | ○ | ○ | ○ | ◉ | ○ | essential |

### Does your paper address subitem 21-i?

Copy and paste relevant sections from the manuscript (include quotes in quotation marks "like this" to indicate direct quotes from your manuscript), or elaborate on this item by providing additional information not in the ms, or briefly explain why the item is not applicable/relevant for your study

Yes. Generalizability was discussed as a limitation. "The study may not be generalizable to all people with diabetes, as tertiary specialist outpatient clinics are likely to manage more complex cases that cannot be managed in the primary care setting."

### 21-ii) Discuss if there were elements in the RCT that would be different in a routine application setting

Discuss if there were elements in the RCT that would be different in a routine application setting (e.g., prompts/reminders, more human involvement, training sessions or other co-interventions) and what impact the omission of these elements could have on use, adoption, or outcomes if the intervention is applied outside of a RCT setting.

| | 1 | 2 | 3 | 4 | 5 | |
|---|---|---|---|---|---|---|
| subitem not at all important | ○ | ◉ | ○ | ○ | ○ | essential |

### Does your paper address subitem 21-ii?

Copy and paste relevant sections from the manuscript (include quotes in quotation marks "like this" to indicate direct quotes from your manuscript), or elaborate on this item by providing additional information not in the ms, or briefly explain why the item is not applicable/relevant for your study

Yes. Physician's recommendation and the data collection during patients follow-up (which was convenient for the patient) influenced the recruitment rates.

## OTHER INFORMATION

## 23) Registration number and name of trial registry

### Does your paper address CONSORT subitem 23? *

Copy and paste relevant sections from the manuscript (include quotes in quotation marks "like this" to indicate direct quotes from your manuscript), or elaborate on this item by providing additional information not in the ms, or briefly explain why the item is not applicable/relevant for your study

No, the trial was not registered as it was originally intended as a pilot study. This was indicated in the manuscript.

## 24) Where the full trial protocol can be accessed, if available

### Does your paper address CONSORT subitem 24? *

Cite a Multimedia Appendix, other reference, or copy and paste relevant sections from the manuscript (include quotes in quotation marks "like this" to indicate direct quotes from your manuscript), or elaborate on this item by providing additional information not in the ms, or briefly explain why the item is not applicable/relevant for your study

The trial protocol is not avilable since this is a pilot study

## 25) Sources of funding and other support (such as supply of drugs), role of funders

### Does your paper address CONSORT subitem 25? *

Copy and paste relevant sections from the manuscript (include quotes in quotation marks "like this" to indicate direct quotes from your manuscript), or elaborate on this item by providing additional information not in the ms, or briefly explain why the item is not applicable/relevant for your study

The study was funded by Nanyang Technological University, Singapore, through (a) the Center for Population Health Sciences (CePHaS), Lee Kong Chian School of Medicine, Nanyang Technological University, Singapore; (b) the Institute for Health Technologies (HealthTech NTU), Nanyang Technological University, Singapore; and (c) Changi General Hospital, Singapore.

## X27) Conflicts of Interest (not a CONSORT item)

### X27-i) State the relation of the study team towards the system being evaluated

In addition to the usual declaration of interests (financial or otherwise), also state the relation of the study team towards the system being evaluated, i.e., state if the authors/evaluators are distinct from or identical with the developers/sponsors of the intervention.

| | 1 | 2 | 3 | 4 | 5 | |
|---|---|---|---|---|---|---|
| subitem not at all important | ○ | ○ | ○ | ○ | ◉ | essential |

### Does your paper address subitem X27-i?

Copy and paste relevant sections from the manuscript (include quotes in quotation marks "like this" to indicate direct quotes from your manuscript), or elaborate on this item by providing additional information not in the ms, or briefly explain why the item is not applicable/relevant for your study

Yes, all authors declared that they have no conflict of interest

## About the CONSORT EHEALTH checklist

### As a result of using this checklist, did you make changes in your manuscript? *

- ○ yes, major changes
- ◉ yes, minor changes
- ○ no

### What were the most important changes you made as a result of using this checklist?

Adding the webpage of the developer in-text

### How much time did you spend on going through the checklist INCLUDING making changes in your manuscript *

6 hours

### As a result of using this checklist, do you think your manuscript has improved? *

- ◉ yes
- ○ no
- ○ Other:

## Would you like to become involved in the CONSORT EHEALTH group?

This would involve for example becoming involved in participating in a workshop and writing an "Explanation and Elaboration" document

- ○ yes
- ◉ no
- ○ Other:

## Any other comments or questions on CONSORT EHEALTH

Your answer

## STOP - Save this form as PDF before you click submit

To generate a record that you filled in this form, we recommend to generate a PDF of this page (on a Mac, simply select "print" and then select "print as PDF") before you submit it.

When you submit your (revised) paper to JMIR, please upload the PDF as supplementary file.

Don't worry if some text in the textboxes is cut off, as we still have the complete information in our database. Thank you!

## Final step: Click submit !

Click submit so we have your answers in our database!

SUBMIT

Never submit passwords through Google Forms.

This content is neither created nor endorsed by Google. Report Abuse - Terms of Service

Google Forms
